# Magnetic field–driven particle assembly and jamming for bistable memory and response plasticity

**DOI:** 10.1126/sciadv.adc9394

**Published:** 2022-11-11

**Authors:** Xianhu Liu, Hongwei Tan, Carlo Rigoni, Teemu Hartikainen, Nazish Asghar, Sebastiaan van Dijken, Jaakko V. I. Timonen, Bo Peng, Olli Ikkala

**Affiliations:** Department of Applied Physics, Aalto University, P.O. Box 15100, FI-02150 Espoo, Finland.

## Abstract

Unlike classic synthetic stimulus-responsive and shape-memory materials, which remain limited to fixed responses, the responses of living systems dynamically adapt based on the repetition, intensity, and history of stimuli. Such plasticity is ubiquitous in biology, which is profoundly linked to memory and learning. Concepts thereof are searched for rudimentary forms of “intelligent materials.” Here, we show plasticity of electroconductivity in soft ferromagnetic nickel colloidal supraparticles with spiny surfaces, assembling/disassembling to granular conducting micropillars between two electrodes driven by magnetic field *B*. Colloidal jamming leads to conduction hysteresis and bistable memory upon increasing and subsequently decreasing *B*. Abrupt *B* changes induce larger conduction changes than gradual *B*-changes. Periodic *B* pulsing drives to frequency-dependent facilitation or suppression of conductivity compared to exposing the same constant field. The concepts allow remotely controlled switching plasticity, illustrated by a rudimentary device. More generally, we foresee adaptive functional materials inspired by response plasticity and learning.

## Teaser

Magnetic field–driven assembly/disassembly of ferromagnetic electroconducting particles shows bioinspired response plasticity.

## INTRODUCTION

Artificial stimulus-responsive materials (*1*–*4*) and shape-memory and shape-morphing materials (*5*–*8*) have already witnessed considerable scientific progress and have been implemented in technologically relevant applications based on their switchable properties. Their properties are, however, limited to fixed states under repeated stimuli, and they do not dynamically adapt or evolve to new states. Recently, it has been pinpointed that there exists an emerging need for more complex adaptive responsivities toward “materials intelligence” and progressively life-like properties (*9*–*13*).

More specifically, in stimulus-responsive materials, the optical, electric, volumetric, transparency, solubility, or wetting properties can be modulated by various stimuli, such as temperature, light, or electric and magnetic fields (*1*–*4*). The representative example is poly(*N*-isopropyl acrylamide) hydrogels, which show volumetric and optical transparency transitions upon temperature changes (*14*). The classic stimulus-responsive materials involve fixed states, and the response always remains the same upon repeated stimuli cycling. In the shape memory materials (*5*–*8*), in the simplest case, there is a second imposed condition or stimulus that allows preparing the material in another kinetically trapped state. This facilitates keeping the materials in the temporary state before releasing them back to the original state by exposing another stimulus. There can even be several temporary states. Again, the properties remain the same upon repeated stimulus cycling. Thus, a generic feature in all of these classic systems is that there are only fixed responses, and the systems do not adapt or evolve upon repeated stimuli.

To search for conceptual ideas for response plasticity in artificial materials, we selected field-driven pillared surface topographies, as topographies are ubiquitous to mediate functions in biology: The aspect ratios of biological topographies vary widely, depending on their functions. Low–aspect ratio hierarchical surface topographies on plant leaves allow liquid and dirt repellency (*15*), whereas high–aspect ratio hairs, cilia, whiskers, trichomes, awns, and antennae enable stickiness, absorption, thermal insulation, debris removal, sensing, and motility (*16*–*23*). The topographies can be (semi)static (*15*, *16*) or dynamic dissipative biological machines (*19*–*23*). Biological surface topographies have indeed inspired the development of functional materials for liquid manipulation, stimulus-responsive wettings, adhesion, and homeostasis by constructing pillars with fixed dimensions but can bend or re-align driven by temperature or external fields (*24*–*32*). However, biological surface topographies generally involve, e.g., growth, assembly, disassembly, memory, adaptation, and evolution.

**Fig. 1.**
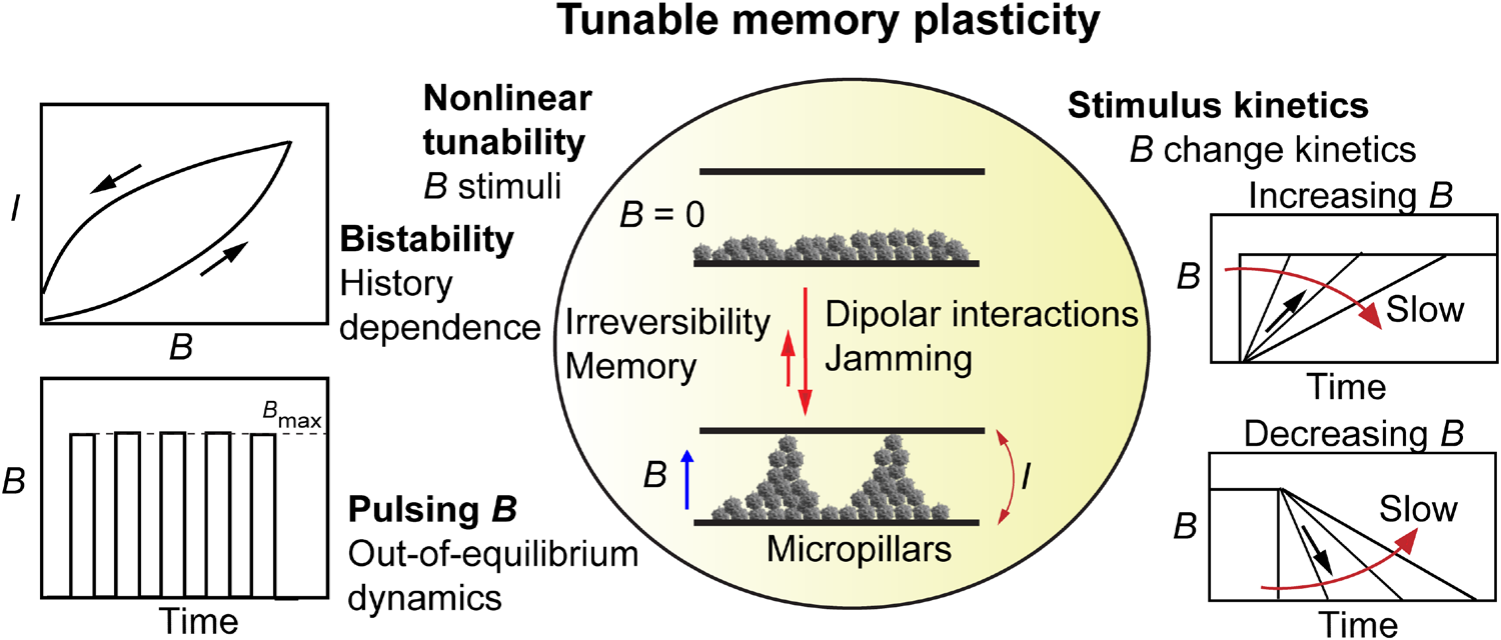
Schemes for tunable, bistable, history-dependent, and pulsing-induced plasticity based on dynamically assembling and disassembling electrically conducting micropillars of SFNCSs based on different magnetic field applications. Bistability of electric current *I* upon increasing and decreasing *B* leads to hysteretic electrical memory due to colloidal jamming. Kinetics of *B* changes lead to history-dependent *I* responses. *B* pulsing leads to spike-tunable electric conductivity based on pulsing frequency and can drive the system out of equilibrium.

In the future, interactive materials, e.g., for emerging soft robotics, magnetic fields, and light will be recognized to be among the few stimuli allowing remote wireless and touchless control (*33*, *34*). Here, we will explore magnetically driven assemblies and disassemblies of electrically conductive pillars as dynamic surface topographies and show how the electric current *I* through them in a circuit responds to the exposed driving magnetic field *B*. The pillars are granular, composed of electrically conducting soft ferromagnetic nickel colloidal supraparticles (SFNCSs). Notably, *I* is highly and nonlinearly tunable by *B*. We will show hysteresis and bistability of *I* upon increasing and reducing *B* based on the jamming of SFNCSs. Jamming is caused by magnetic dipolar attractions among particles, potentially also promoted by their spiny surfaces, thus leading to hysteretic structural memories. Colloidal jamming has previously been considered, e.g., in liquid-liquid interfaces (*35*), but not for electrical bistability, plasticity, and reconfigurable memory. On the other hand, hysteretic memory and jamming in the mechanical properties of metamaterials have recently been presented using interlocked ring-like components, corrugated sheets, or three-dimensional printed tileable elements (*36*–*38*). In our case, abrupt *B* changes lead to larger *I* changes than the gradual *B* changes do, suggesting transitions between kinetically trapped structures. We demonstrate the plasticity under magnetic field pulsing, where *I* is either facilitated or suppressed by the repeating *B* pulses, depending on the pulsing frequency, compared to the current allowed by a constant *B* (see Fig. 1 for schematics). In the end, we will translate, to the simplest form, the response plasticity into touchless dynamic adaptive switching of a light-emitting diode array.

## RESULTS

### Synthesis and characterization of SFNCSs

**Fig. 2.**
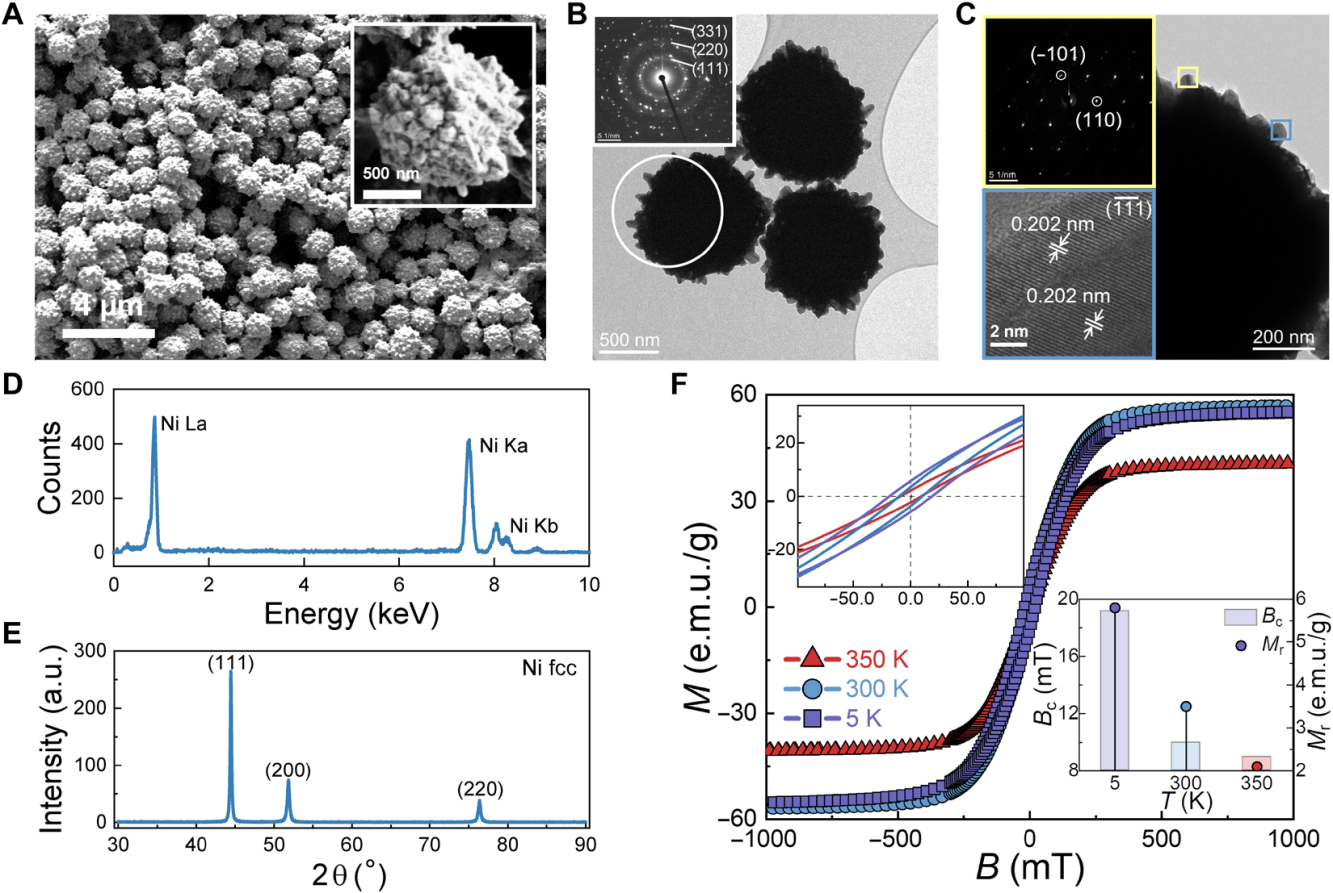
Characterization of SFNCSs. (**A** to **C**) Scanning (A) and transmission (B and C) electron micrographs. Insets: The high-magnification electron micrograph in (A) depicts the spiny surface and (C) shows the fcc ($\bar{111}$) lattice fringes; the selected area electron diffraction characterization in (B) and (C) reveals that the particles are clusters of single-crystalline nanocrystals. (**D** to **E**) Energy-dispersive x-ray spectroscopy (D) and x-ray diffraction (E) confirm the nickel nature of the particles. a.u., arbitrary unit. (**F**) Temperature dependence of the magnetization *M* as a function of *B*. The decline of saturation magnetization *M*_s_ from 300 to 350 K may be related to crystal transformation (fig. S7). The insets show a high-magnification image in the range from −100 to 100 mT (top left) and a summary of temperature dependence of the coercivity *B*_c_ and the remanence *M*_r_ (bottom right). e.m.u., electromagnetic unit.

The SFNCSs are solvothermally synthesized using NiCl_2_ precursor, reduced at 200°C in the presence of NaOH in ethylene glycol (EG) (also see Materials and Methods). They are stabilized with trisodium citrate (fig. S1), and the micrometer-sized particles consist of nanograins (Fig. 2, A to C) and have spiny surfaces (fig. S2 and table S1). Electron diffraction shows supraparticles comprising face-centered cubic (fcc) single-crystalline nickel nanograins (Fig. 2, B and C, insets). These results are corroborated by energy-dispersive x-ray spectroscopy (Fig. 2D), x-ray photoelectron spectroscopy (fig. S3), x-ray diffraction (Fig. 2E), and thermal gravimetry with a nickel content of 98.7 weight % (fig. S4). The SFNCSs are soft ferromagnetic up to 350 K (Fig. 2F and fig. S5). Further temperature increase will lead to superparamagnetism (*39*).

The morphological and magnetic properties of the SFNCSs are tunable by the synthesis temperature (figs. S6 and S7 and table S2). Elevating the temperature from 160° to 200°C increases the mean size from 0.5 to 1.2 μm, the grain size from 24.2 to 62.6 nm, and the magnetization from 10.8 to 56.8 electromagnetic unit (e.m.u.)/g, respectively. Further increasing the reaction temperature to 250°C reduces the magnetization because of crystal transformation to the hexagonal close-packed phase and consequently particle’s size. The ferromagnetic nature retains irrespective of the synthesis temperature, differing from the previous results where superparamagnetic samples were usually obtained (*40*).

### Magnetically driven assembly, disassembly, and jamming of SFNCSs

**Fig. 3.**
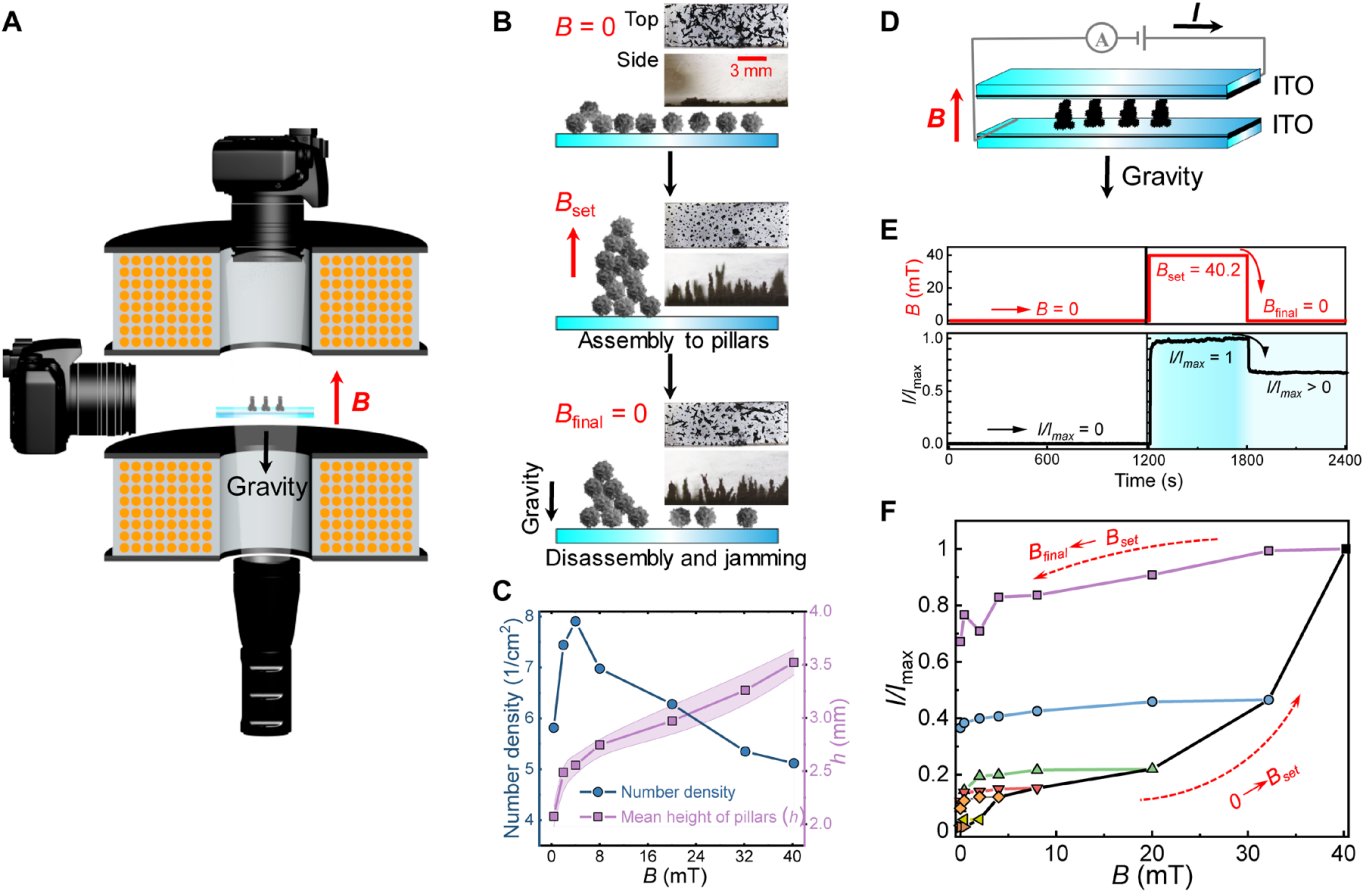
Magnetic field–driven nickel colloidal supraparticle assembly, partial disassembly, and jamming to micropillar topographies for tunable and bistable hysteretic current response. (**A**) Schematics of the experimental setup used for investigating *B*-driven assembly of the SFNCSs under a pair of Helmholtz coils that induce homogeneous magnetic fields antiparallel to the gravity. (**B**) *B*-driven assembly, partial disassembly, and jamming of the SFNCSs on a glass substrate, and the top and side views of the micropillars. (**C**) Unconstrained heights and number density of the pillars on a glass substrate as a function of increasing *B*. (**D**) SFNCSs sandwiched between two indium tin oxide (ITO)–coated glass slides that are connected as a circuit. The distance between the ITO-coated glasses is 1.2 mm. (**E**) An example of *B*-driven electrical currents mediated by the assembly of pillars. In the absence of *B*, no current flows. Upon exposing the maximum available field *B*_max_ = 40.2 mT, the maximum current *I*_max_ is achieved, and upon removing *B*, a residual current *I/I*_max_ ≈ 0.7 is observed, indicating bistability. (**F**) Imposing different magnetic fields *B*_set_ shows that the current *I/I*_max_ is nonlinearly tunable by the magnetic field, and subsequently, its reduction to *B*_final_ displays the current hysteresis and bistability.

The *B* driven assembly of SFNCSs to granular pillared surface topographies is studied using a pair of Helmholtz coils allowing the application of homogeneous perpendicular fields (Fig. 3A and fig. S8). SFNCSs, spread on a glass plate, initially show no pillared topographies without *B* (Fig. 3B, top). However, they assemble into micropillars upon exposing *B* antiparallel to gravity (Fig. 3B, middle) because of the induced magnetic dipole-dipole interactions between the particles that become preferentially aligned along the *B* direction (fig. S9). A large number of shallow pillars are observed at *B* < 4.0 mT on an underlying glass substrate, but fewer and higher pillars form upon further increasing *B* (Fig. 3C). Eventually, the pillar height reaches ~3.5 mm at the presently available maximum field *B*_max_ = 40.2 mT (fig. S10). Upon removing *B*, the pillars slightly collapse (Fig. 3B, bottom). However, they do not totally collapse because of jamming, thus facilitating a hysteretic structural memory function. The residual jammed structures have been visualized by scanning electron microscopy, as shown in fig. S11, after the partial collapse upon the removal of the *B*_max_ = 40.2 mT. The areal density is 73.2% with an SD of 1.3%, agreeing with the previous findings in other systems and the theoretical results (*41*–*44*).

We next explore the dependence of the current *I* on the magnetic field *B*. The inherent electrically conductive nature of the SFNCSs allows in situ characterization of the assembly/disassembly of the pillars. Thus, SFNCSs, as sandwiched between two indium tin oxide (ITO)–coated glass slides to form a circuit (Fig. 3D), show initially *I* = 0 at *B* = 0. However, switching *B* to *B*_max_ = 40.2 mT quickly during a fraction of a second, *I* first increases steeply, followed by a slow stabilization thereof (Fig. 3E and fig. S12, A and B). The largest available constant field, i.e., *B*_max_ = 40.2 mT, leads to the largest available current; therefore, this current is denoted as *I*_max_ (fig. S12). Subsequent quick switching *B* to zero leads to residual current *I*/*I*_max_ ≈ 0.7 (Fig. 3E) as the pillars only partly disassemble because of colloidal jamming (Fig. 3B, bottom). Therefore, hysteresis, i.e., bistability, is observed, allowing triggerable electrical memory instead of complete reversibility. In general, nonlinear dependence of *I* on the set magnetic field *B*_set_ is observed (Fig. 3F and fig. S12). For small *B*_set_ up to ca. 4.0 mT, *I* increases steeply as pillars initially form (agreeing with Fig. 3C), suggesting the percolation of the initially formed pillars between the electrodes. Thereafter, a less steep plateau is observed. In contrast, a higher current is observed at even higher *B* due to the increased effective pillar contact areas with the ITO-coated electrodes (fig. S13) induced by the enhanced magnetic normal pressure (fig. S14). The currents can be tuned widely by varying the magnitude *B*, the particle content (fig. S12), and the gap between the electrodes.

To explore the conductivity bistability for different fields, we subsequently reduced *B* to *B*_final_ (0 mT ≤ *B*_final_ < *B*_set_) to record the residual *I/I*_max_ (Fig. 3F and figs. S15 and S16). The residual *I/I*_max_ values vary as a function of the applied *B* values. This suggests a tunable memory effect and hysteretic bistability, where the bistability in *I/I*_max_ is smaller for lower *B*_final_ (fig. S17). Decreasing the difference between *B*_set_ and *B*_final_ or increasing the duration of *B*_set_ substantially increases the residual *I/I*_max_ (figs. S18 and S19), signifying plasticity. It also indicates that prolonging the application of *B* field facilitates the jamming and the current. Otherwise, the residual *I/I*_max_ decreases or even vanishes as a substantial difference is applied with only a short duration of *B*_set_ (fig. S19B).

The assembly/disassembly hysteresis allowing the electrical bistability involves complexity of interactions. The field-dependent magnetic dipolar attractions are evaluated (*45*) in the Supplementary Materials for SFNCSs synthesized at different temperatures (fig. S20). These interactions promote the *B* driven particle assembly to micropillars. By contrast, the gravitational forces promote their disassembly. Figure S20F shows that the latter interactions are ca. three orders of magnitude smaller, underpinning the importance of the magnetic interactions for the stabilization and jamming. We suggest that the particle surface roughness may further promote the stability of the micropillars. The roughness with ca. 50-nm surface spikes (fig. S2 and table S1) can promote the interparticle friction under the magnetic dipolar interactions. The *B* dependent dipolar forces could then also explain that it is easier to assemble the pillars at low fields than to disassemble them at high fields. Also, as suggested by studies of sandpile falls, the particle roughness may further promote the stability of the pillars if atmospheric water could be confined within the roughness (*46*). The jammed structures formed at higher *B* values can be reset by mechanical shaking, and therein, the residual current recovers back to zero and the memory is erased.

### Kinetic tuning of the *B* driven bistable memory

**Fig. 4.**
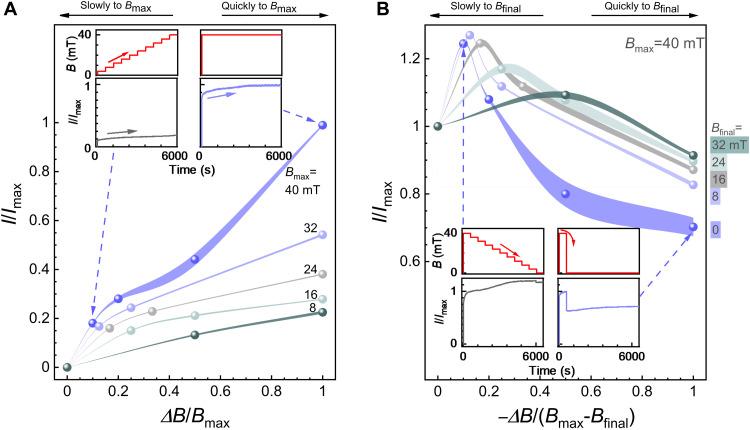
Electric current responses upon different magnetic field change kinetics. (**A**) Achievable current as a function of field step sizes (∆*B*) up to different maximum magnetic field values *B*_max_, where, e.g., ∆*B*/*B*_max_ = 1 means that *B* is switched to *B*_max_ quickly in one step and ∆*B*/*B*_max_ = 0.1 that 10 steps are used. A quick increase of *B* from 0 to *B*_max_ in a fraction of a second renders higher current *I* than gradually increasing the field to the same value using multisteps. The current is expressed as *I/I*_max_, where *I*_max_ corresponds to the highest available current obtained by the highest available *B* = 40.2 mT. (**B**) Residual currents upon first assembling the pillars at *B*_max_ = 40.2 mT (corresponding to *I*_max_) followed by a quick or a gradual decrease of the field to *B*_final_ in step sizes of ∆*B*. The linewidths indicate error bars.

Subsequently, we compare the effects of quick and gradual *B* switching on the current, respectively. First, a rapid single-step increase to the maximum *B*_max_ = 40.2 mT within a fraction of a second allows for the stabilized current, denoted as *I*_max_ (used for subsequent scaling, unless specified otherwise; see the right inset in Fig. 4A). By contrast, a slow gradual stepwise *B* increase to the same *B*_max_ during 6000 s leads to an order of magnitude lower *I/I*_max_ (Fig. 4A, left inset) than that of a quick increase. More generally, the resulting *I/I*_max_ upon increasing the field to different *B*_max_ using different step-size ∆*B* is shown in Fig. 4A and fig. S21. They show that large step-size ∆*B*, i.e., quick *B* changes, lead to large current responses.

Next, the micropillar disassembly is explored. The residual *I/I*_max_ is investigated after the micropillars have first been assembled at *B*_max_ = 40.2 mT, subsequently reducing the field to different *B*_final_ quickly or gradually at step-size ∆*B* (Fig. 4B). If the field is reduced quickly to *B*_final_ (within a fraction of a second), then the residual current *I/I*_max_ ≈ 0.7 is observed for *B*_final_ = 0 due to colloidal jamming (Fig. 3E). By contrast, if the field is reduced to zero gradually, i.e., slowly, by (−∆*B*/(*B*_max_ − *B*_final_) = 0.1, i.e., 10 steps, then the *I/I*_max_ is unexpectedly not reduced, as it even increases slightly instead. This increase is subtle and might be caused by the structural annealing, i.e., particle rearrangements, under the lower *B* fields. The general behavior is shown in Fig. 4B, from 40.2 mT to different *B*_final_, using different ∆*B* (Fig. 4B and fig. S22).

A common conclusion in both cases is that the current values and their changes depend on the magnetic field and its history. In particular, large abrupt *B* changes render large *I* changes. As shown in fig. S19B, structural annealing under high fields over a long time span reinforces the particle jamming. In contrast, rapid *B* changes offer less time for annealing than slow *B* changes do, suppressing jamming and thus increasing *I* changes and the final current (fig. S23). The responses are nonlinear, reconfigurable, and more adaptive to different states than the responses in the classic stimuli-responsive (*1*–*4*) or shape-memory (*5*–*8*) materials, where, characteristically, switching between fixed states/phases are observed.

### Magnetic field pulsing–driven plasticity

**Fig. 5.**
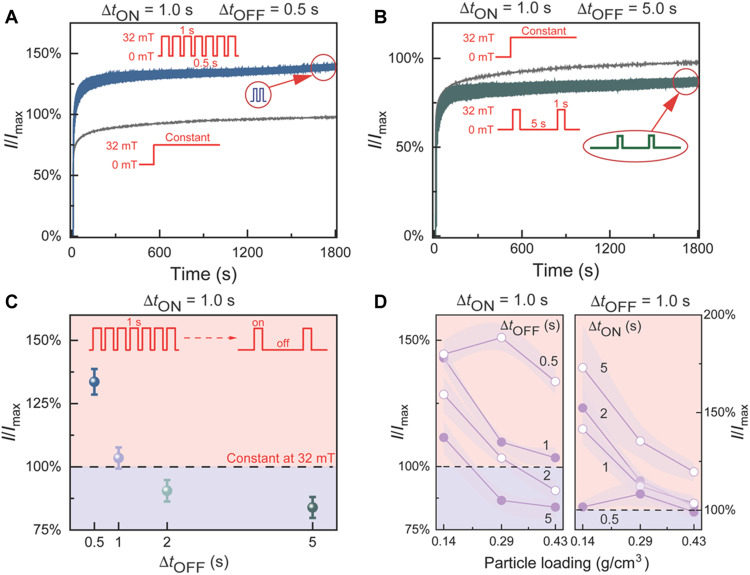
Current plasticity upon magnetic field pulsing. (**A**) An example of pulsing induced facilitation in *I*/*I*_max_ using Δ*t*_ON_ = 1.0 s, short Δ*t*_OFF_ = 0.5 s, and pulsing up to *B*_max_ = 32 mT that leads to higher current than that by applying the dc field at *B*_max_. Note that the *I*_max_ is defined in this case as the highest current obtained using the dc field at this *B*_max_. The resulting pulsing of the current manifests as the thicker line, where the individual current oscillations are not resolved in this time scale. (**B**) An example of pulsing induced suppression using Δ*t*_ON_ = 1.0 s, long Δ*t*_OFF_ = 5.0 s, and pulsing up to *B*_max_ = 32 mT. (**C**) Transition from facilitation to suppression as a function of increasing Δ*t*_OFF_ for fixed Δ*t*_ON_ = 1.0 s. In (A) to (C), the loading of SFNCS within the electrode volume corresponds to 0.43 g/cm^3^ for the total SFNCS weight versus the available confined volume between the electrodes. (**D**) *I*/*I*_max_ plasticity as a function of the loading of SFNCS within the electrode volume for different values of Δ*t*_ON_ and Δ*t*_OFF_ (see also fig. S26).

Next, we demonstrate the plasticity of the current response upon exposing periodic magnetic field pulses, inspired by the short-term plasticity of cellular scale neuronal spiking or macroscale psychological behavioral changes due to repeated stimuli (*47*). As a reference, we use here the application of the dc magnetic field to *B*_max_ = 32 mT, whereupon the dc reaches its corresponding maximum value defined in this case as *I*_max_ for this field value [Fig. 5 (A and B) and shows the time evolution of the scaled dc in black thin lines]. This *I*_max_ is used to scale *I* also for the pulsed cases. Important for the subsequent results is that upon applying *B*_max_, there is first a rapid current change in seconds, followed by subsequent slow change, due to the micropillar assembly kinetics (fig. S12). Next, pulsing magnetic field is used upon switching *B* ON to *B*_max_ for the time Δ*t*_ON_, followed by switching *B* OFF to zero field for the time Δ*t*_OFF_. This pulsing is repeated using the values Δ*t*_ON_, Δ*t*_OFF_ = 0.5, 1.0, 2.0, and 5.0 s. These times have been selected being on the same order as the major assembly/disassembly kinetics of the micropillars (figs. S12 and S18). As expected, the current values oscillate in synchrony with the *B*-pulses. However, because of the selected values of Δ*t*_ON_ and Δ*t*_OFF_, the micropillars and the currents do not completely relax but oscillate dynamically at tunable values. For example, for Δ*t*_ON_ = 1.0 s and Δ*t*_OFF_ = 0.5 s, *I*/*I*_max_ shows 36% higher value upon *B*-pulsing than using the dc field at *B*_max_ (Fig. 5A), i.e., the *B* pulsing facilitates the conductivity. However, for Δ*t*_ON_ = 1.0 s and Δ*t*_OFF_ = 5.0 s, *I*/*I*_max_ becomes lower than that of the dc field at *B*_max_ (Fig. 5B), i.e., the *B* pulsing suppresses the conductivity. More systematically, Fig. 5C shows that keeping Δ*t*_ON_ = 1.0 s, the *I*/*I*_max_ response changes systematically from a “facilitating” to “suppressing” one upon increasing Δ*t*_OFF_, directly demonstrating an intriguing form of current plasticity. Another parameter controlling the pulsing dynamics of the micropillars deals the selected total amount of SFNCS, denoted here by its total mass versus the total available volume defined between the electrodes (g/cm^3^). Thereof, three amounts were explored, i.e., 0.43, 0.29, and 0.14 g/cm^3^. Figure 5D shows *I*/*I*_max_ as a function of these particle loadings for different Δ*t*_ON_, Δ*t*_OFF_, upon keeping Δ*t*_ON_ = 1.0 s fixed (left) and keeping Δ*t*_OFF_ = 1.0 s fixed (right). Expectedly, the general trend is that higher dynamic levels of *I*/*I*_max_ are obtained for Δ*t*_OFF_ < Δ*t*_ON_. Also, to allow the promoted plasticity, a large amount of SFNCS are needed to facilitate the initial particle jamming, leaving sufficient flexibility for the subsequent structural optimization by *B* pulsing from suppression to facilitation. Although conceptually and mechanistically different, this artificial plasticity by repeating exposing stimuli could be put qualitatively in the concept of short-term synaptic plasticity in nervous systems or habituation/sensitization in macroscale behavior (*47*). Also, we suggest that the dynamical plasticity is achieved by driving the system out of equilibrium by *B*-pulsing.

### Magnetic remote control involving plasticity

**Fig. 6.**
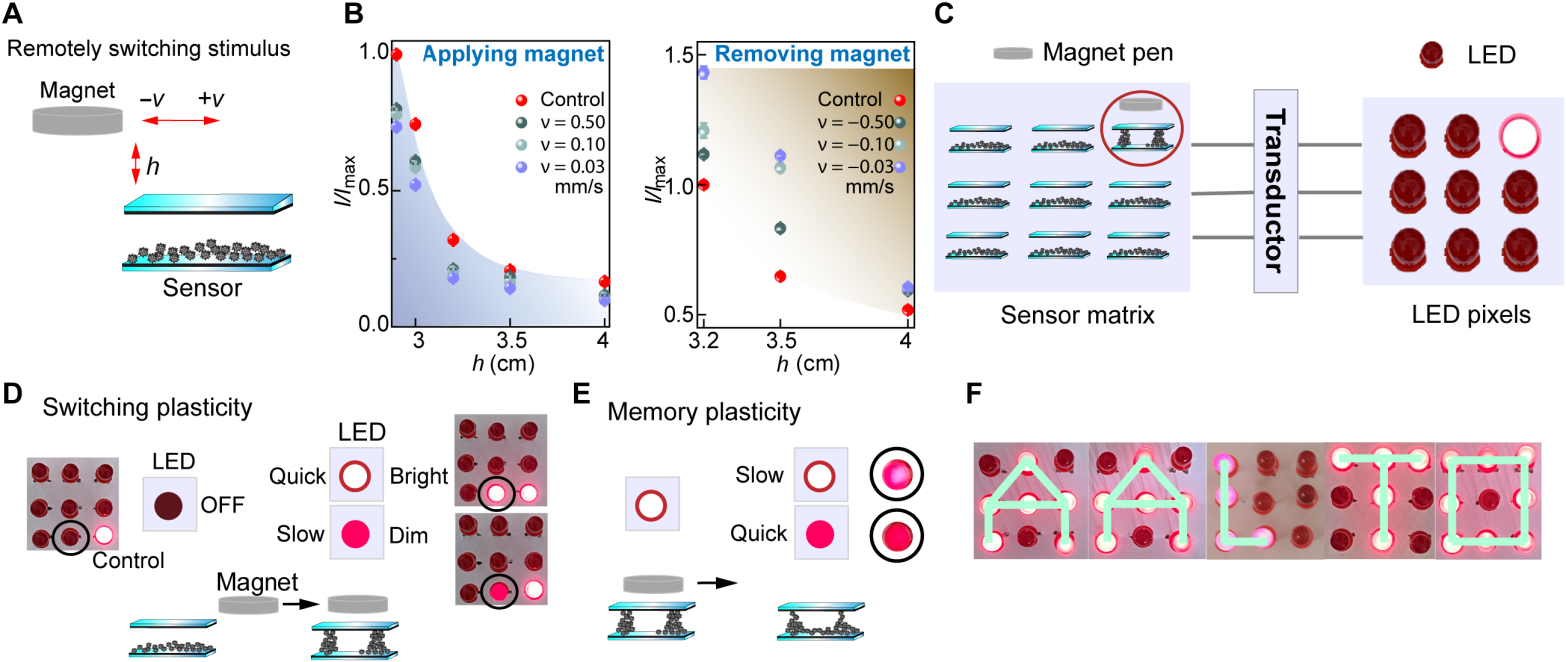
Demonstration of remotely controlled switching plasticity. (**A**) Schematics of switching plasticity based on remotely controlled magnetic field stimulus, determined by the distance from the sensor element (*h*) and magnetic application kinetics (*v*). (**B**) Quantification of the current response in the sensor element based on different *h* and *v*. Control group (red): manually quickly placing the magnet pen to the given sensor element. (**C**) Scheme of the sensor and LED matrices, as rudimentary magnetically switchable pixels. (**D**) Scheme of OFF/ON plastic switching based on different application velocities of the magnetic pen. (**E**) Schematic design of the memory plasticity, where different levels of LED brightness are resolved upon different magnet removal velocities from the sensor element. (**F**) Demonstration of touchless writing of the AALTO by facilitation process (movie S10).

Magnetic field allows remotely controlled touchless and wireless stimulus application, which opens new options for interactive materials and soft robotics (*48*, *49*). The above findings suggest that magnetic field strength and application kinetics are crucial in the dynamical assembly/disassembly control of the conducting micropillars, allowing remotely controlled plasticity in the electrical response (Fig. 6, A and B). As a rudimentary demonstration, we construct a 3 × 3 light-emitting diode (LED) system, where the magnetic stimulus for each LED pixel is controlled using a remotely applied “magnetic pen” applied for the corresponding sensory element based on a neodymium magnet at different distances and speeds toward or away from the corresponding sensor element (Fig. 6C). Although the applied absolute *B*-field is different to prior experiment, the tunability of the current plasticity is qualitatively similar. For instance, a slow approach toward a sensor element leads to a weaker LED brightness than the rapid approach, i.e., suppressive adaptation (Fig. 6D, movies S5 to S7, and figs. S27 and S28). Also, slowly moving the magnetic pen away from the corresponding sensor retains the brightness (Fig. 6E and movies S8 and S9), i.e., facilitative adaptation (fig. S29). These results verify that the current plasticity can be achieved even with commercial magnets, promising portable applications. In the end, we demonstrate the touchless writing of the “AALTO” on this panel by facilitation process.

## DISCUSSION

In summary, we have shown a one-pot solvothermal synthesis of electrically conducting SFNCSs, which allow magnetic field–driven assembly and disassembly of micropillars with large nonlinear tunability of electrical current *I* and the hysteresis for bistability based on jamming. The increase and decrease of magnetic field induce asymmetry in the micropillar assembly/disassembly, which reflects in conduction, resulting in conduction bistability. Abrupt *B* changes in comparison to slow *B* changes facilitate large current changes, signaling history dependence. Applying pulses of magnetic field leads to dynamically tunable facilitated or suppressed current response in comparison to the exposure of the continuous magnetic field, inspired by biological plasticity. The *B* pulsing may also drive the system out of equilibrium. The findings suggest promising concepts for the development of life-like concepts using magnetic fields, which are suggested to allow remote controls for interactive materials, such as for remotely controlled soft robots.

## MATERIALS AND METHODS

### Materials

Sodium hydroxide pellets (NaOH; ≥98.0%), sodium citrate tribasic dihydrate (NaCit; ≥99.0%), and nickel(II) chloride (NiCl_2_; 98.0%) were obtained from Sigma-Aldrich. Ethanol (≥99.5%) was purchased from Altia Oyj, Finland. EG (≥99.0%) was provided by Thermo Fisher Scientific. Deionized water (18.2 megohm) was used in all experiments and obtained from a Millipore Direct-Q UV 3 reverse osmosis filter apparatus. All chemicals were used as received.

### Preparation of SFNCSs

The synthesis is based on a solvothermal polyol method and the reduction of a nickel precursor (NiCl_2_) in EG in the presence of NaOH and NaCit. Typically, 1.6 g of NiCl_2_ and 1.8 g of NaCit were dissolved in 30 ml of EG by magnetic stirring at 150°C to form a neon green solution. In parallel, 1.8 g of NaOH was dissolved in 30 ml of EG at 150°C to yield a dark brown solution. Subsequently, the as-prepared NaOH/EG solution was mixed with the NiCl_2_/NaCit/EG phase. The mixture was sealed in a 100-ml autoclave, heated at 200°C for 15 hours, and then naturally cooled to room temperature. In the end, the product was collected by a permanent magnet and washed with ethanol and water repeatedly to remove the retained solvent. The SFNCSs were lastly stored in a nitrogen glove box after vacuum drying at room temperature until use in the subsequent experiments.

### Design of device

A pair of Helmholtz coils (GMW 3470) were used to generate a magnetic field opposite to gravity. The coils were powered by a multirange dc power supply (BK Precision 9205), and their generated magnetic fields were detected and calibrated with a Gauss meter (LakeShore 410). The sample powder was sealed and sandwiched between two glass slides that are interiorly coated with ITO. This device was placed in between the two coils, where the homogeneous magnetic fields can be generated (fig. S8). The external magnetic field enables the formation of magnetic pillars that bridge the two ITO coatings, allowing for the closure of a circuit. This circuit was connected with an electrometer/high resistance meter (Keysight B2987A), by which 1 V was applied, and the current (*I*) was measured simultaneously. The in situ observation of the magnetic pillars was carried out with a digital single-lens reflex camera (Nikon D5500). Mechanical shaking of the experimental cells allows the assembled pillar structure to collapse, resetting the system to its initial state.

## Supplementary Materials

### This PDF file includes:

Supplementary Text Figs. S1 to S29 Tables S1 to S3 References
