# Supplementary material for: Magnetic field–driven particle assembly and jamming for bistable memory and response plasticity
Source: Sci Adv. 2022 Nov 11;8(45):eadc9394. doi: 10.1126/sciadv.adc9394 (PMC9651856; doi:10.1126/sciadv.adc9394)
Supplement: Supplementary file 1 — Supplementary Text Figs. S1 to S29 Tables S1 to S3 References [file sciadv.adc9394_sm.pdf]

Science Advances
AAAS

Supplementary Materials for

Magnetic field–driven particle assembly and jamming for bistable memory and response plasticity

Xianhu Liu *et al.*

Corresponding author: Bo Peng, pengbo006@gmail.com; Olli Ikkala, olli.ikkala@aalto.fi

Sci. Adv. **8**, eadc9394 (2022)
DOI: 10.1126/sciadv.adc9394

The PDF file includes:

Supplementary Text
Figs. S1 to S29
Tables S1 to S3
Legends for movies S1 to S10
References

Other Supplementary Material for this manuscript includes the following:

Movies S1 to S10

SUPPLEMENTARY TEXT

CHARACTERIZATION METHODS

Electron microscopy (SEM and TEM)

A Zeiss Sigma VP scanning electron microscope (SEM) operated at 10 kV was used to analyze the morphology for a global overview of the SFNCSs. A JEOL JEM-2200FS transmission electron microscope (TEM) with an accelerating voltage of 200 kV was used to study the detailed morphology, the size, the energy dispersive X-Ray spectroscopy (EDX), and electron diffraction of the products. To allow SEM and TEM characterization, a droplet of aqueous particle suspension with proper concentrations was cast onto silica wafers and carbon-coated copper grids, respectively, allowing the solvent to evaporate at room temperature. The polydispersity (*Pd*) of SFNCSs is defined as

$$Pd(\%) = \frac{\sigma}{\bar{d}}, \quad (1)$$

where $\bar{d}$ is the mean size of the particles and σ is the standard deviation of the sizes.

X-ray diffraction (XRD)

XRD patterns of the powder samples were recorded with a Rigaku SmartLab X-Ray diffractometer using Cu$K\alpha$ radiation (λ = 0.15406 nm) at 35 kV and 15 mA to analyze the crystal structures at a scanning rate of 0.5° min^{-1} using the 2θ angle range from 30° to 90°. The average grain size can be estimated with the Debye-Scherrer formula:

$$\tau = \frac{K\lambda}{\beta cos\theta}, \quad (2)$$

where τ is the mean size of the ordered (crystalline) domains, K is the dimensionless shape factor with a typical value of 0.89 for spherical particles, λ is the X-ray wavelength, β is the width of the XRD peak at the half-peak height in radians, and θ is the Bragg angle.

Superconducting quantum interference device (SQUID) magnetometry

The magnetic properties and susceptibilities were measured with a Quantum Design MPMS-XL7 SQUID magnetometer at 5, 300, and 350 K with an applied field between -1000 and 1000 mT. For zero-field-cooled (ZFC) and field-cooled (FC) measurements, the samples were initially cooled in a zero field to 5 K. With an applied field of 10 mT, the susceptibility was recorded as ZFC curves by increasing the temperature. After the temperature had reached 400 K, the samples were progressively cooled and the susceptibility was recorded as an FC curve.

Fourier-transform infrared spectroscopy (FTIR)

FTIR was measured using PerkinElmer Spectrum Two FT-IR Spectrometer in the wavenumber region of 500-4000 cm^{-1} with dry samples using sample mass ca. 0.1 g.

X-ray photoelectron spectroscopy (XPS)

XPS was measured using a Kratos Axis Ultra device for investigating the surface components of the SFNCSs (elemental concentration and chemical state). CasaXPS was used to analyze the data.

Thermogravimetric analysis (TGA)

TGA was performed with a TA Instruments Q500 Thermogravimetric Analyzer by heating the sample from room temperature to 900 ^{o}C with a rate of 5 ^{o}C/min under air atmosphere.

SUPPLEMENTARY FIGURES, TABLES, AND ANALYSES

Characterization of SFNCSs

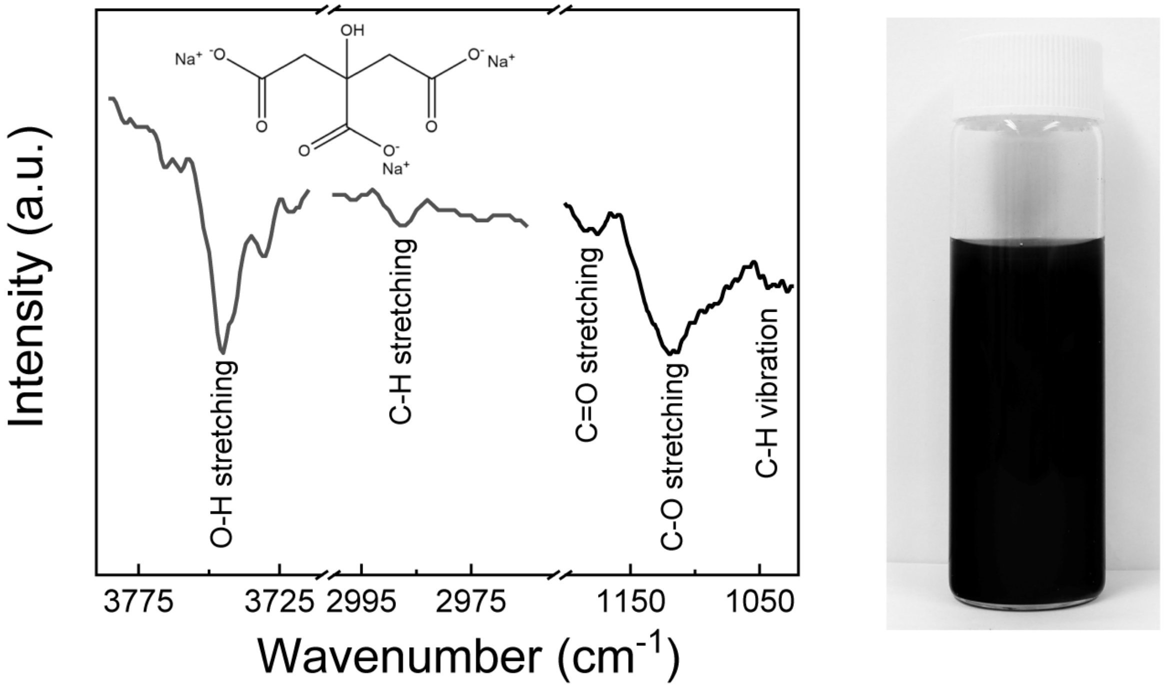

Fig. S1. FTIR spectrum of trisodium citrate (NaCit) stabilized SFNCSs synthesized at 200 ºC and a photograph of aqueous SFNCSs dispersion. The characteristic peaks correspond to NaCit as identified in the FTIR spectrum.

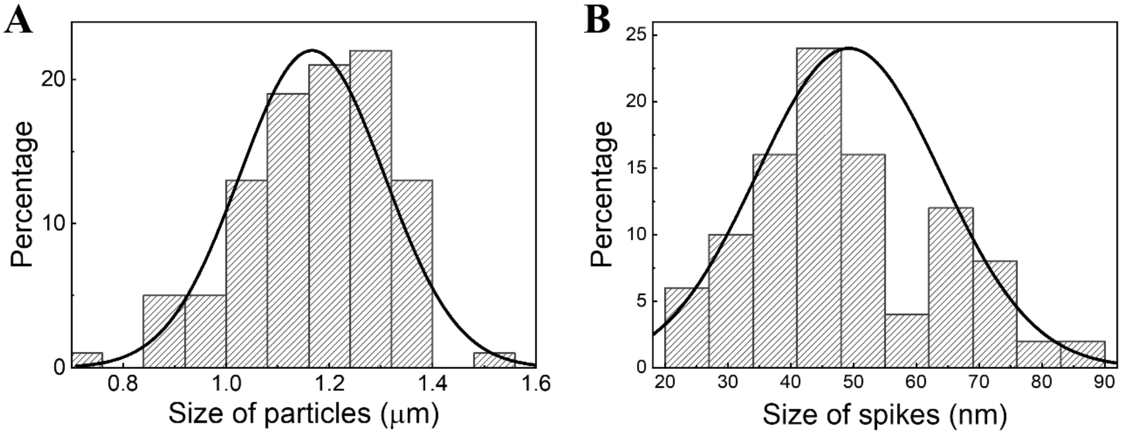

Fig. S2. Particle and surface spike size distributions of the SFNCSs synthesized at 200 ºC. Size statistics of the particles (**A**) and their surface spikes (**B**). The details are summarized in table S1.

Table S1. The size summary of SFNCS particles synthesized at 200 °C and their surface spikes.

	$\bar{d}$	σ	Pd (%)
Particles	1.2 μm	0.1 μm	8.3
Spikes	49.3 nm	14.7 nm	29.8

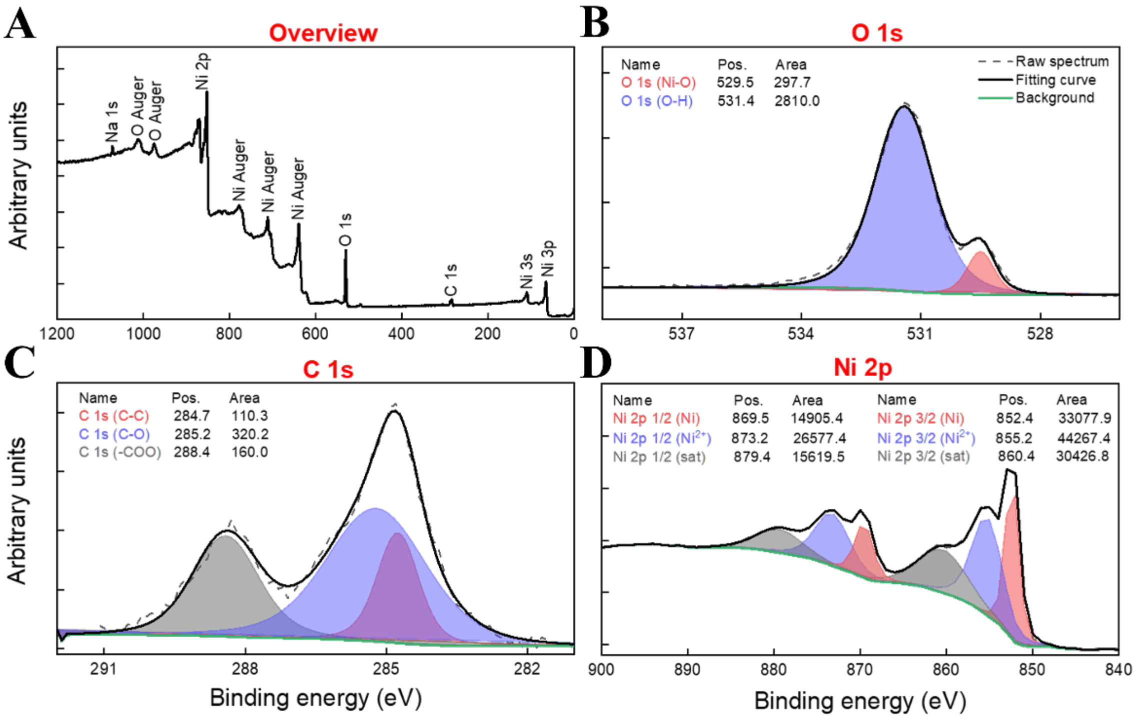

Fig. S3. XPS spectra and analysis of SFNCSs synthesized at 200 °C. (**A**) The overview of the spectrum from 0 to 1200 eV. (**B**) Elemental identification of O1s from NaCit, EG, and NiO. (**C**) Elemental identification of C1s from C-C, C-O and -COO bonds, which are ascribed to NaCit and EG. (**D**) Elemental identification of Ni 2p from zero-valent nickel and Ni^{2+}.

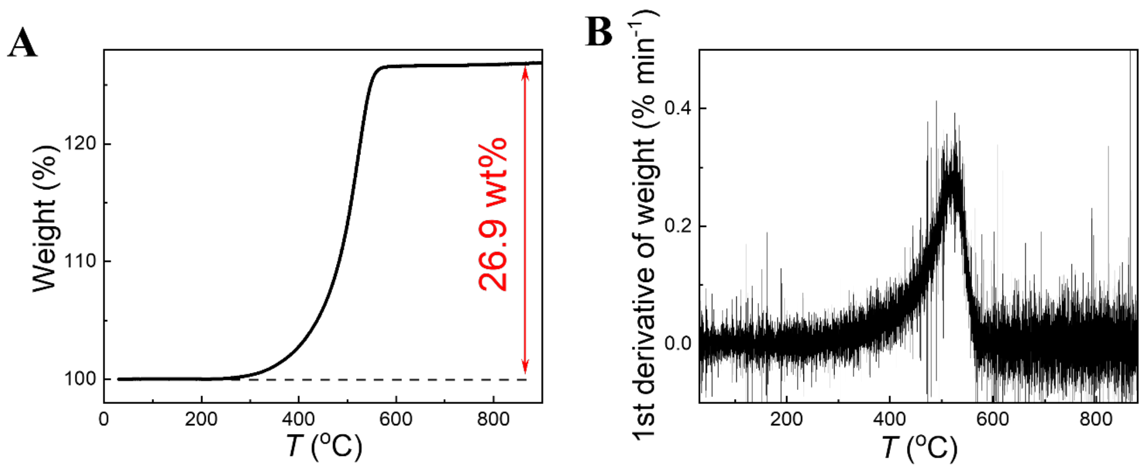

Fig. S4. TGA analysis of SFNCSs (synthesized at 200 ºC) in air. (**A**) TGA results: the oxidation of nickel starts at ca. 270 ºC. The weight gain is ca. 26.9 wt% which is close to the theoretical value of 27.3 wt% for pure nickel. This suggests that the nickel content of SFNCSs is 98.7 wt%. (**B**) The first derivative of the weight variation from the data in (**A**).

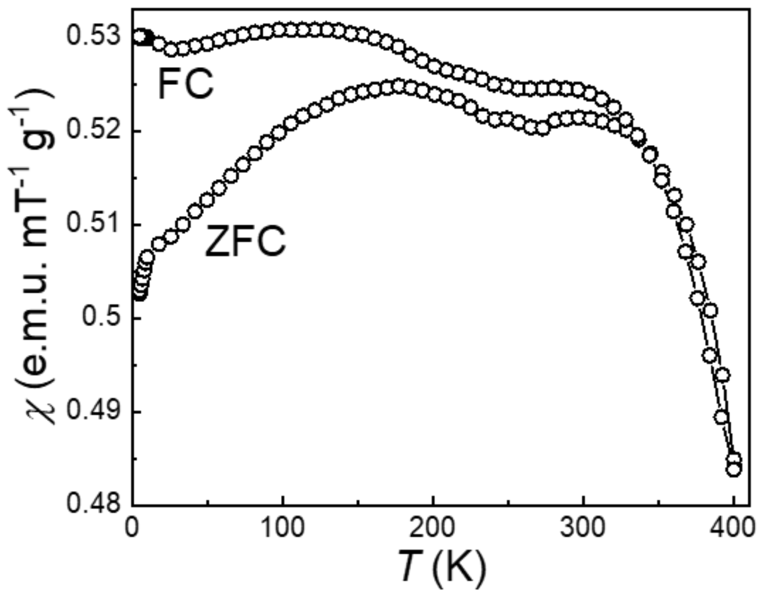

Fig. S5. Magnetic field cooling (FC) and zero-field cooling (ZFC) measurement of SFNCSs synthesized at 200 ºC.

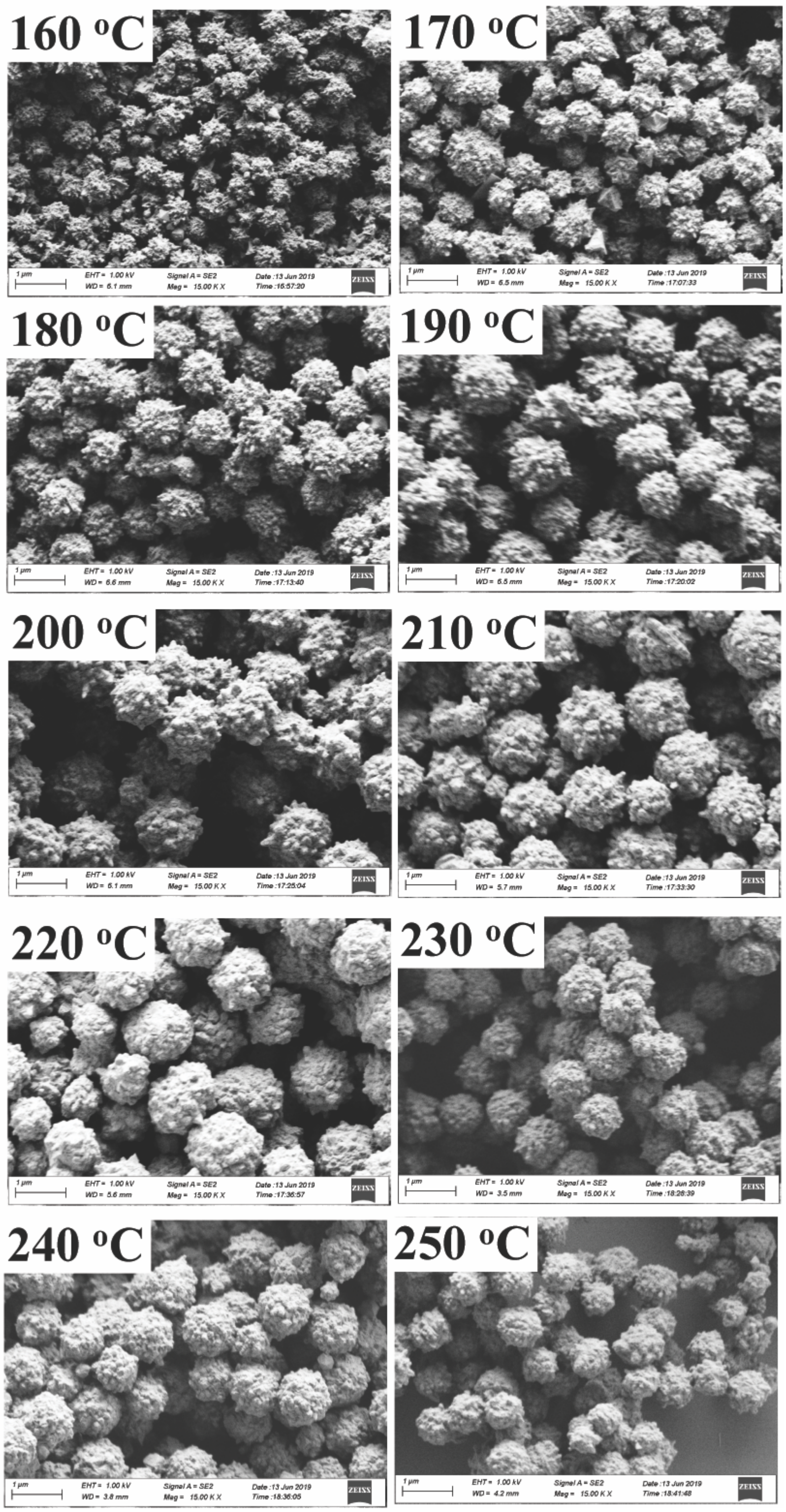

Fig. S6. Morphology tuning of SFNCSs by altering the reaction temperature from 160 to 250 °C. The size details are summarized in fig. S7B and table S2.

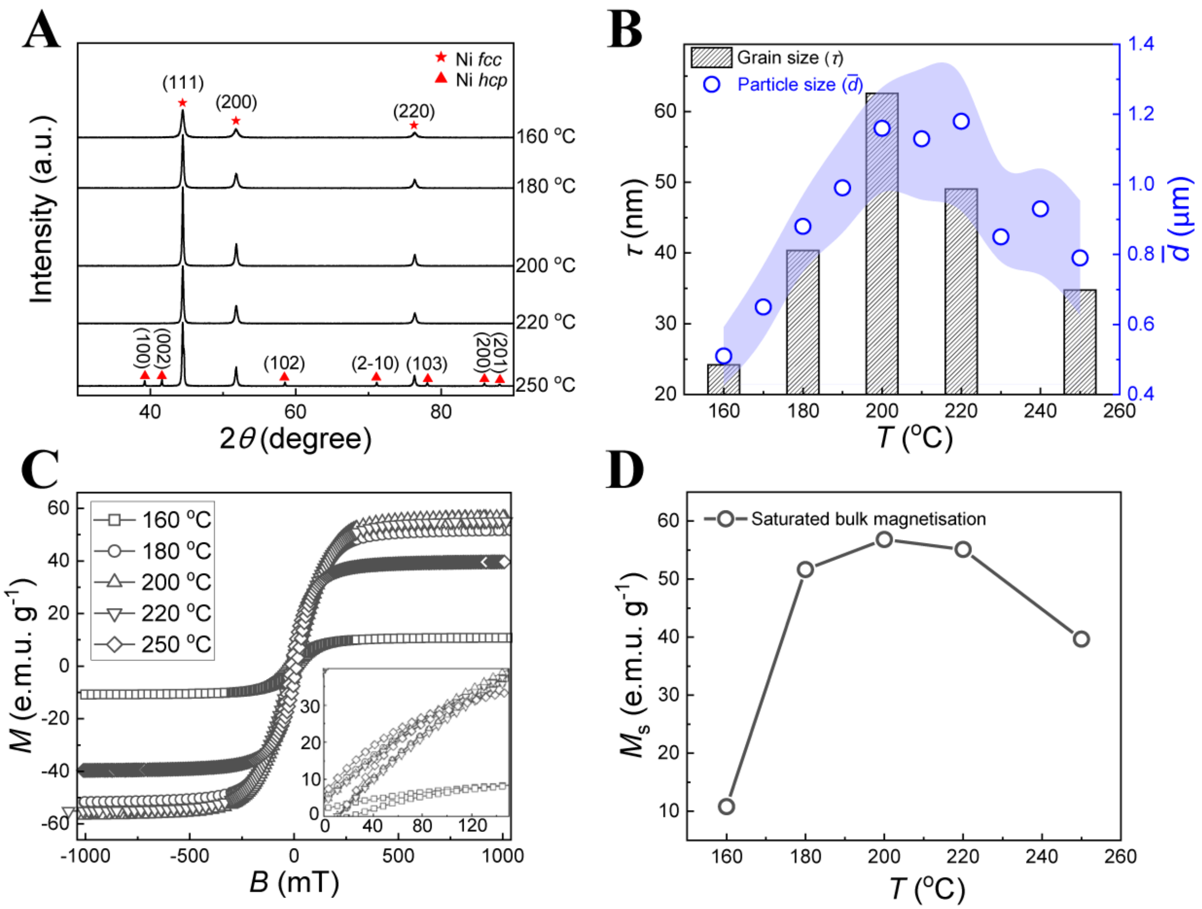

Fig. S7. The effect of the reaction temperature on the crystal structures and magnetic properties of the SFNCSs. (A) XRD patterns, (**B**) statistics of the particle and grain sizes (summarized from figs. S6 and S7A), and (**C**) the mass magnetic moment *M* and and the saturated bulk magnetization M_s. Inset: *M* in the range of 0-150 mT. (**D**) M_s for SFNCSs synthesized under 160, 180, 200, 220, and 250 ^{o}C. Note that the blue shadow in (B) indicates the standard deviations.

Table S2. A summary of the average particle size $\overline{d}$, grain size τ, saturated mass magnetic moment M_s, coercivity B_c, and the remanence M_r of the SFNCSs synthesized using different reaction temperatures T.

T (ºC)	$\overline{d}$ (μm)	τ (nm)	M_s (emu/g)	B_c (mT)	M_r (emu/g)
160	0.5	24.2	10.8	21.2	2.0
180	0.9	40.4	51.6	12.2	4.5
200	1.2	62.6	56.8	10.0	3.5
220	1.2	49.1	55.1	10.6	3.6
250	0.8	34.8	39.7	12.6	5.9

Magnetic field characterization of the electromagnet

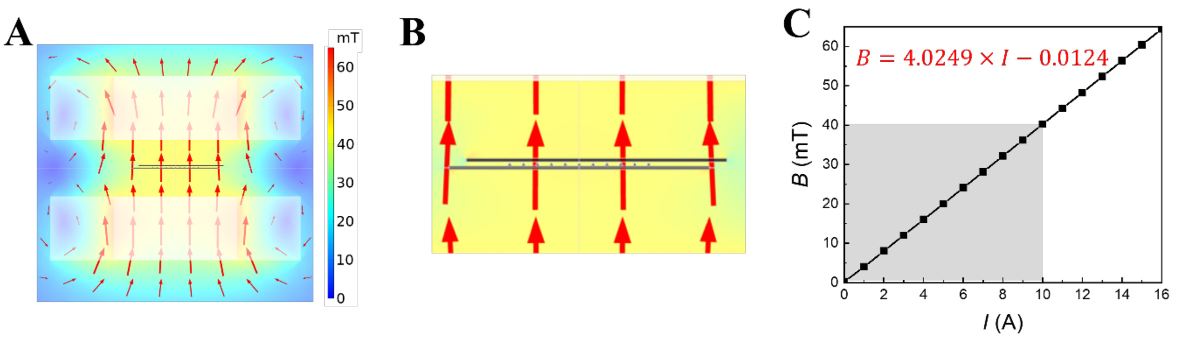

Fig. S8. Magnetic field profile. (**A**) The simulated magnetic fields generated by a pair of Helmholtz coils, (**B**) the magnified field profile illustrated around the sample site, and (**C**) the fields measured with a Gauss-meter. The exposed magnetic fields on the sample site are homogeneous and linearly proportional to the electric current applied.

Magnetic dipolar interactions

The induced magnetic dipolar-dipolar interaction between two particles can be estimated using:

$$F_m(l,\mu_1,\mu_2) = \frac{3\mu_0}{4\pi l^4}\{(\hat{l}\times\mu_1)\times\mu_2 + (\hat{l}\times\mu_2)\times\mu_1 - 2\hat{l}(\mu_1\cdot\mu_2) + 5\hat{l}[(\hat{l}\times\mu_1)\cdot(\hat{l}\times\mu_2)]\}, \quad (3)$$

where μ_0 is the magnetic constant, μ_i is the relative magnetic permeability of the particle i, l is the distance between two magnetic dipoles, and $\hat{l}$ is the unit vector of the centre line of the two dipoles. When the magnetic permeabilities of the two magnetic dipoles are identical, Eq. 3 can be simplified as:

$$F_m(l,m_p,\theta) = \frac{3\mu_0 m_p^2}{4\pi l^4}[2\cos\theta + 1 - 5\cos^2\theta], \quad (4)$$

where the m_p is the magnetic moment of the particles, θ is the angle between the direction of the external magnetic field and the center line of two magnetic dipoles.

The dipolar-dipolar interaction between two SFNCSs is plotted as a function of l and θ, as shown in fig. S9. In the presence of magnetic fields, the particles favor the head-to-tail alignment along the external magnetic field direction rather than the side-by-side alignment, which drives the assembly of the SFNCSs to micropillars.

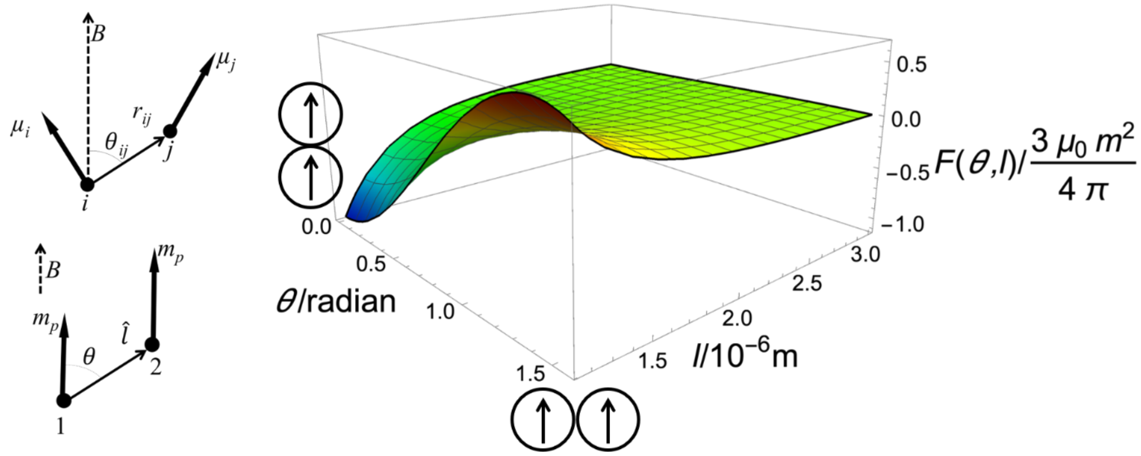

Fig. S9. The magnetic dipole-dipole interaction analysis of SFNCSs in the presence of magnetic fields.

Assembly and jamming of SFNCSs on a single underlying substrate

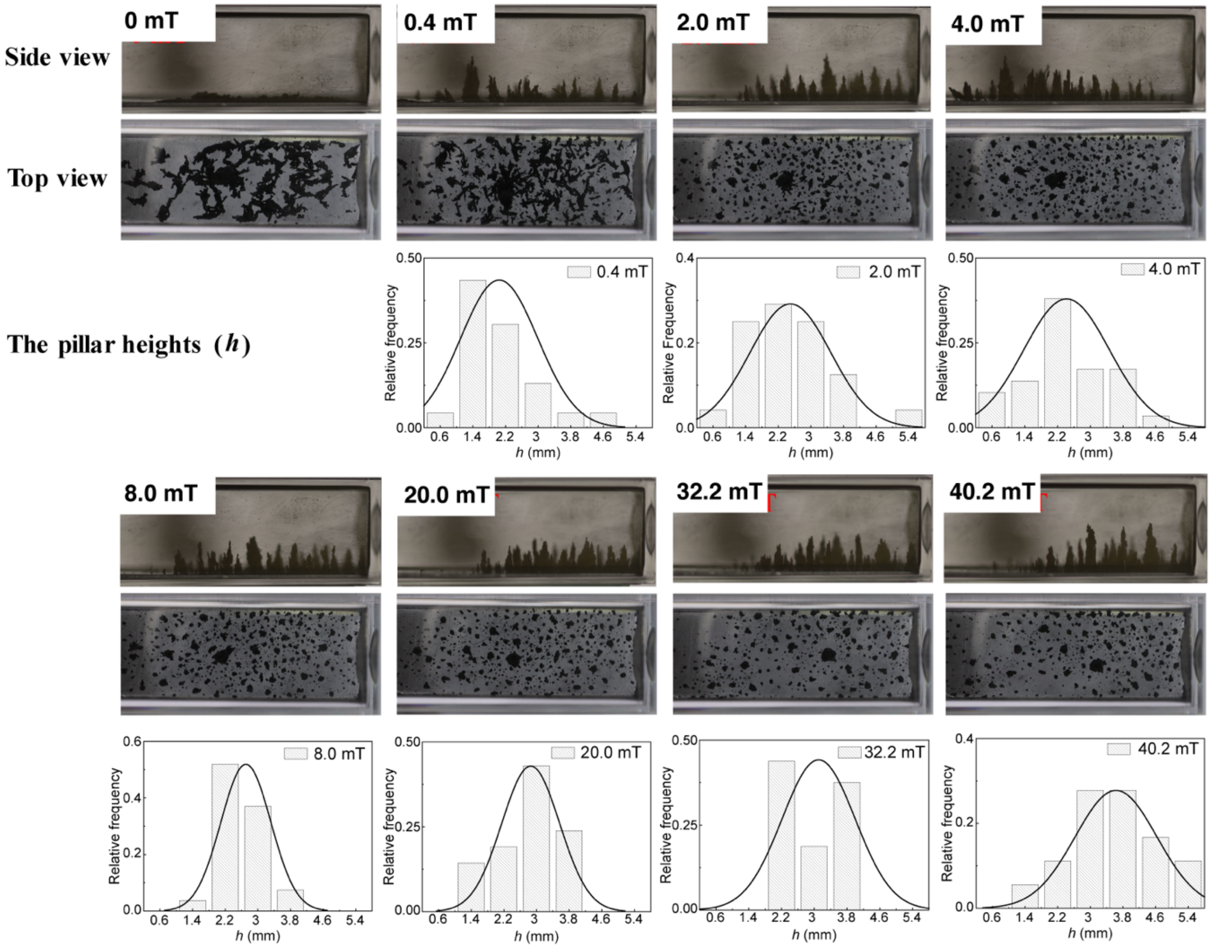

Fig. S10. The unconstrained *B*-driven assembly of SFNCSs to micropillars on an underlying substrate without the upper electrode. The side and top views and the statistical height histograms of SFNCS micropillars based on the side view images upon magnetic field increase of $0 \rightarrow B_{\mathrm{set}}$. Note that there can be a slight initial nonuniformity of the SFNCS loading on the substrate, leading to minor initial height nonuniformity of less than 0.2 mm, which is negligible in comparison to the pillar heights upon exposing the magnetic fields.

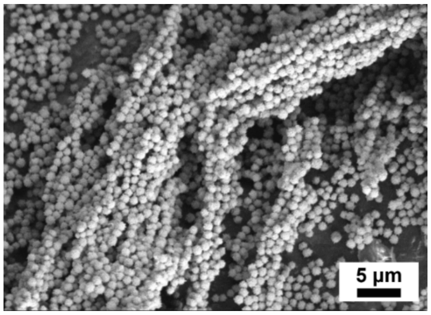

Fig. S11. SEM overview of residually jammed pillars. The SEM overview of residually jammed pillars after collapse upon removing the 40.2 mT magnetic field.

Electric characterization of SFNCS assembly between two electrodes

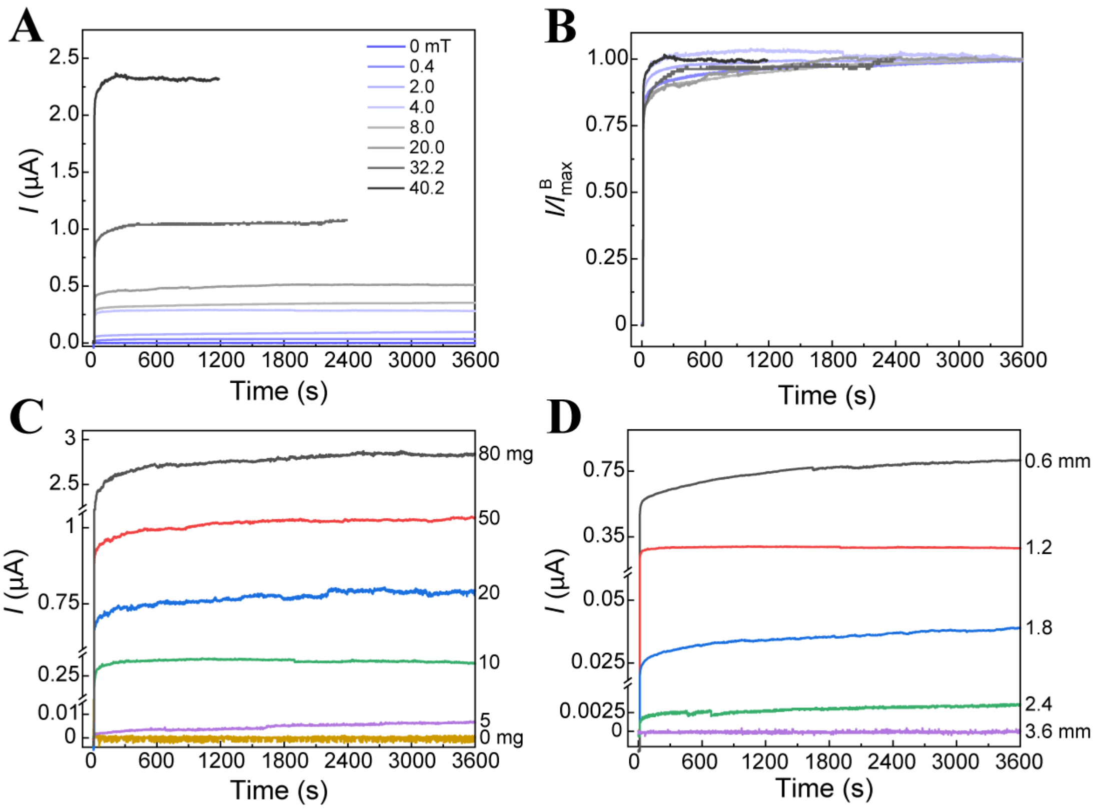

Fig. S12. Electric currents across assembled SFNCS micropillars between two electrodes upon applying a voltage of 1 V. (**A**) The electric current across assembled magnetic pillars as a function of magnetic field strength. (**B**) The normalized *I* as a function of time under the magnetic fields ranging from 0.4 to 40.2 mT, where in this specific case I_{max}^{B} corresponds to the maximum current at the the corresponding different magnetic fields *B*. (**C**) The effect of sample loading between the electrodes on *I*. In these experiments *B* is 8.0 mT and the distance *d* between the two electrodes is 1.2 mm. (**D**) The effect of *d* on *I*. 10 mg of the sample loadings are used and *B* = 8.0 mT. The effective contact area is 1.44 cm^2 between the electrodes with a separation of 1.2 mm.

Estimation of the available current based on the micropillar shape

Here we aim to rationalize the current values depending on the micropillar shape. We first evaluate the average current through one micropillar at 8 mT and suggest generalization thereafter. The unconfined pillar shapes are approximated to be conical, see fig. S10, having average height h = 2.8 mm and bottom radius R = 0.4 mm at 8 mT. Suppose that the micropillars between two electrodes with separation d can be approximated to be truncated cones, see schematically in fig. S13A. Then, the average volume of one truncated conical micropillar is:

$$v_{one\ pillar} = \frac{1}{3}\pi R^2 (h - \frac{(h-d)^3}{h^2}), \tag{5}$$

and the average mass $m_{\text{one pillar}}$ is:

$$m_{one\ pillar} = \frac{1}{3}\rho\varepsilon\pi R^2 (h - \frac{(h-d)^3}{h^2}), \tag{6}$$

where the density of solid nickel ρ = 8.9 g/cm^3 and ε is the filling fraction of spherical particles within the micropillars. Taken that we aim to estimate the maximum current, we assume the ideal packing, corresponding to ε =0.74. In practice, it is expected to be much less.

In fig. S12D, the total mass of applied SFNCSs is m_{total} = 10 mg. Therefore, the effective number of micropillars N_{eff} can be approximated to be $N_{\text{eff}} = m_{\text{total}}/m_{\text{one pillar}}$, i.e.,

$$N_{eff} = \frac{3m_{total}}{\rho\varepsilon\pi R^2 (h - \frac{(h-d)^3}{h^2})}, \tag{7}$$

Next, we take the current values I_{d} of fig. S12D for different electrode separations d = 0.6, 1.2, 1.8, 2.4, and 3.6 mm. The micropillar arrays can be considered to be resistors in parallel. Therefore, the average current in one micropillar is estimated to be $I_d N_{eff}$.

Taken the truncated conical cylinder model at 8 mT, each micropillar has the contact area $S = \pi R^2 (h-d)^2/h^2$ to the upper electrode, which is smaller than the contact area πR^2 to the lower electrode. The current density of each micropillar through the upper contact area $I_d N_{eff}/S$ is a characteristic value for each electrode separation d, see table S3 and fig. S13B, showing values in the range $I_d N_{eff}/S \approx 1 - 18$ µA/mm^2. At 8 mT, the micropillars are conical. As we have already assumed the ideal packing of the particles, the effect of increasing the magnetic field remains to change the conical shape of the micropillars to be ultimately cylindrical ones. In this case, the upper contact surface area S would be the same as the lower one, i.e., $S = \pi R^2$ = 0.50 mm^2 where we can use the value R = 0.4 mm. This would allow an estimation of the maximum current density at high fields, being in the range of 29 µA/mm^2.

Table S3. Evaluation of the micropillar conductivity properties based on figs. S10d S12.

d (mm)	S (mm^2)	I_d (μA)	Current density through one micropillar $I_d N_{eff}/S$ (μA/mm^2)
0.6	0.31	0.83	18.7
1.2	0.16	0.28	7.0
1.8	0.064	0.039	2.4
2.4	0.010	0.0036	1.4

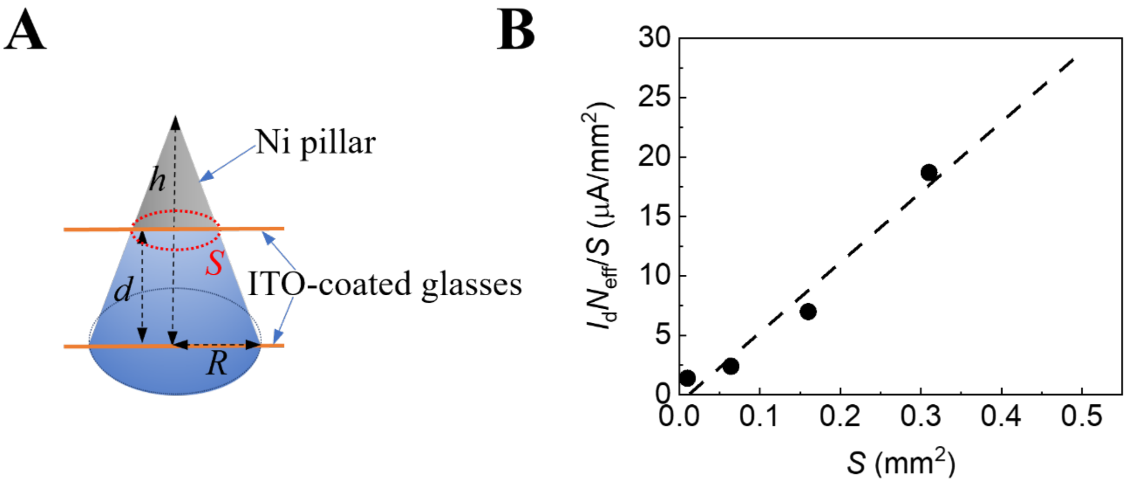

Fig. S13. The relationship between the assembled micropillar shapes and the current. (**A**) The schematics of a SFNCS micropillars placed between two electrodes, where h and R are the height and the bottom radius of the unconstrained pillar, respectively. (**B**) The current density through one micropillar $I_d N_{eff}/S$ as a function of the average contact area S of one pillar to the upper electrode.

Magnetic normal pressure

The low-field part (0-60 mT) of the mass magnetization in fig. S7C can be linearly fitted as shown in fig. S14A. The slopes of the linear fittings suggest the magnetic susceptibility of the samples. Considering the SFNCSs powder as a continuum, we can define the magnetic normal pressure (*50*) similarly as has been done for magnetic liquids. Therefore, P_n can therefore be estimated using Eq. 7:

$$P_n = \frac{1}{2}\mu_0 M^2, \quad (8)$$

where M is the magnetization at given external magnetic field strengthen B, calculated based on the linear fittings in fig. S14A. The relation between the magnetic normal pressure P_n and h is shown in fig. S14B (see also Fig. 3C).

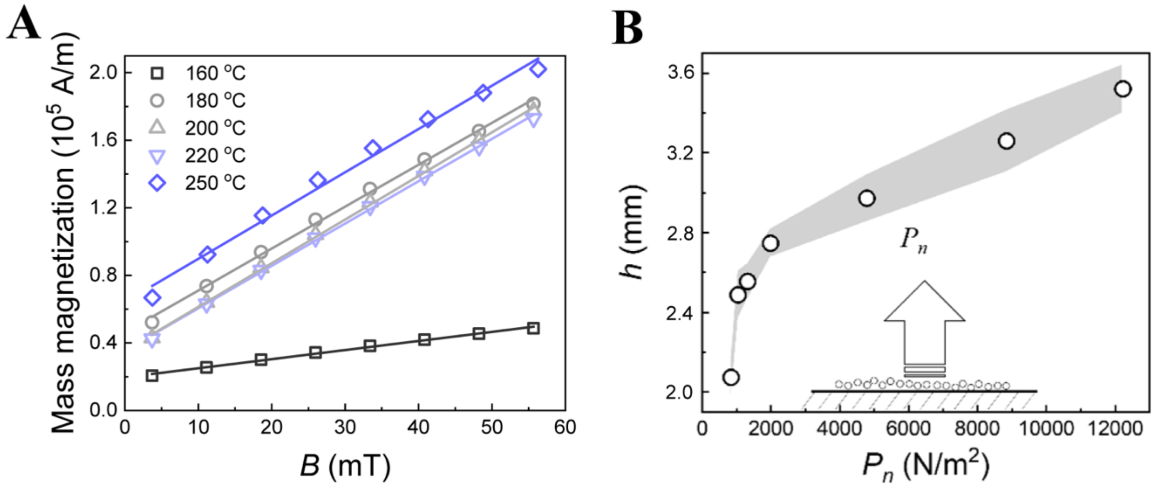

Fig. S14. The characterization and analysis of magnetic field induced magnetic properties of SFNCSs. (**A**) The low-field part 0-60 mT of the mass magnetization of SFNCSs and their linear fittings. (**B**) The relation between the magnetic normal pressure P_n and h.

Disassembly of magnetic pillars between two electrodes by reducing the magnetic field

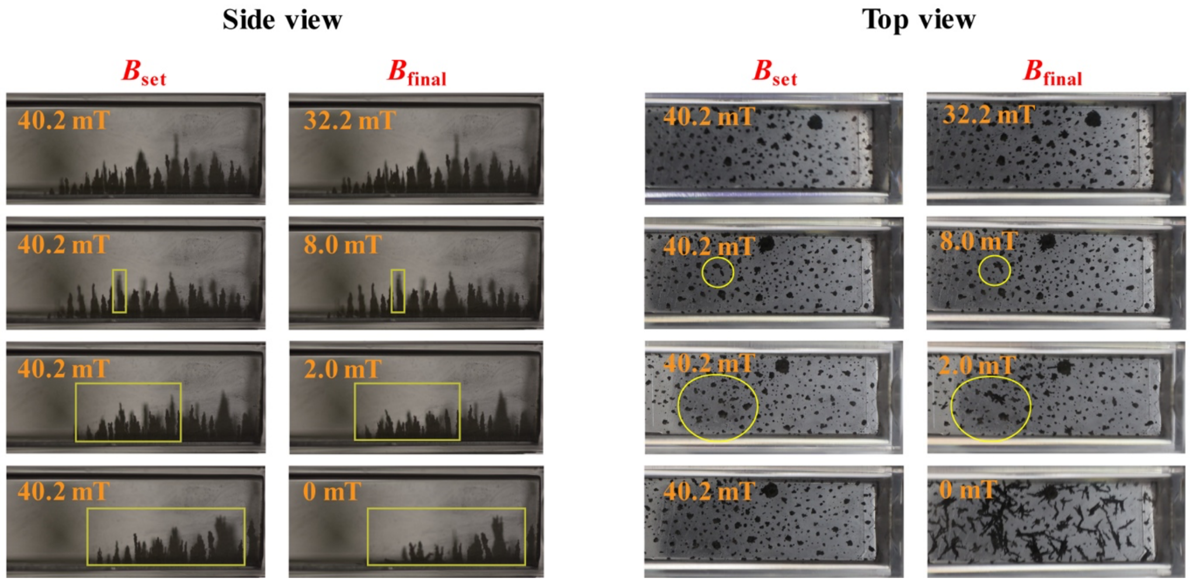

Fig. S15. The unconstrained B-driven disassembly of SFNCSs on an underlying substrate without the upper electrode. Side and top optical views of magnetic micropillars as $B_{\mathrm{set}} = 40.2$ mT → B_{final} (32.2-0 mT).

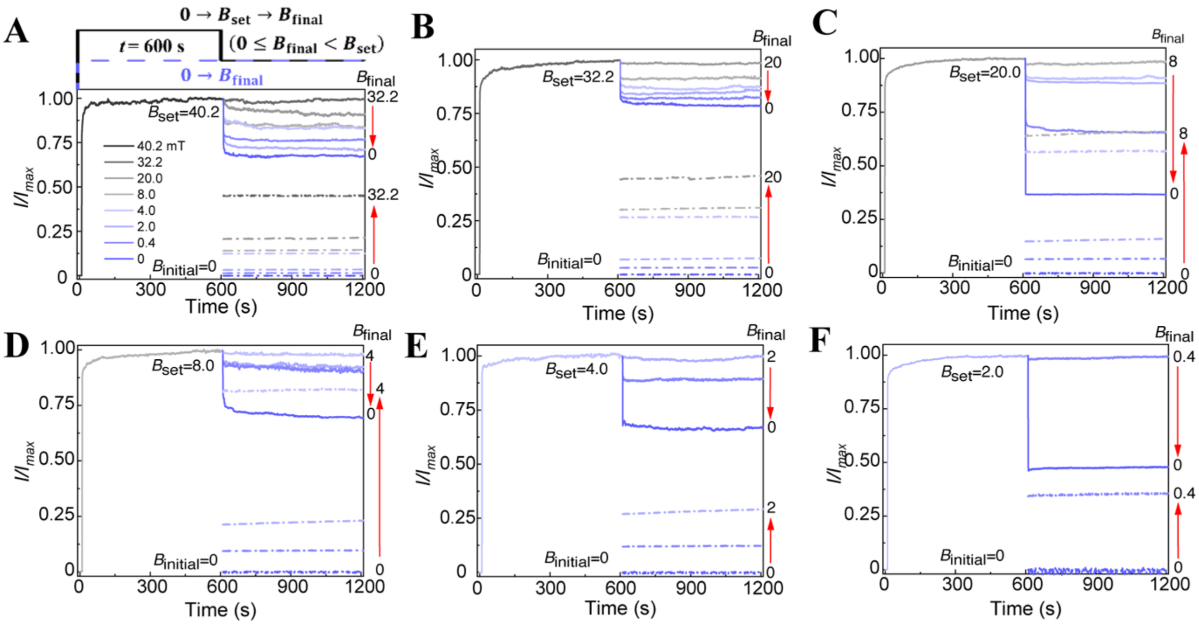

Fig. S16. The current across the pillars between two electrodes based on an initial assembly and subsequent disassembly. (**A** to **F**) The disassembly of magnetic micropillars by decreasing the magnetic field from B_{set} = 40.2 (A), 32.2 (B), 20.0 (C), 8.0 (D), 4.0 (E) and 2.0 mT (F) to B_{final} ($0 \rightarrow B_{set} \rightarrow B_{final}$), respectively, in comparison to those acquired using directly by $0 \rightarrow B_{final}$ (the dashed lines). Effects of B_{final} are depicted with color codes in all cases, specified in (A). I_{max} is the current corresponding to the maximum available field of the electromagnet 40.2 mT.

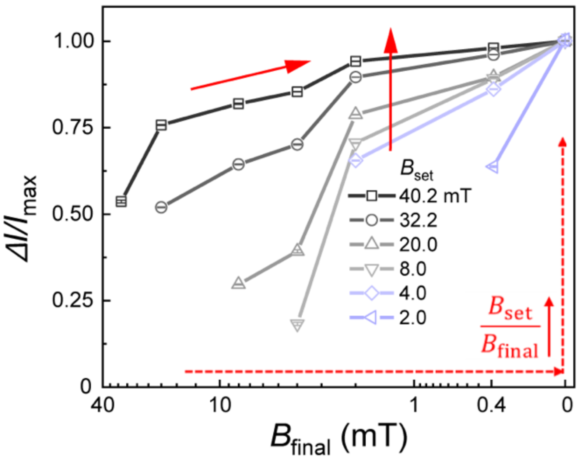

Fig. S17. The relationships between normalized current changes $\Delta I/I_{max}$ and B_{final}. Relationships between normalized $\Delta I/I_{max}$ where ($\Delta I = \frac{I_{B_{final},(0\rightarrow B_{set}\rightarrow B_{final})} - I_{B_{final},(0\rightarrow B_{final})}}{I_{B_{final},(0\rightarrow B_{set}\rightarrow B_{final})}}$) and B_{final} for different B_{set}. For a given B_{set} or B_{final}, $\Delta I/I_{max}$ increases with increasing the ratio B_{set}/B_{final}, indicating that the residual current increases upon exposure to high B_{set}, followed by reducing to lower B_{final}. I_{max} is the current corresponding to the maximum available field of the electromagnet 40.2 mT.

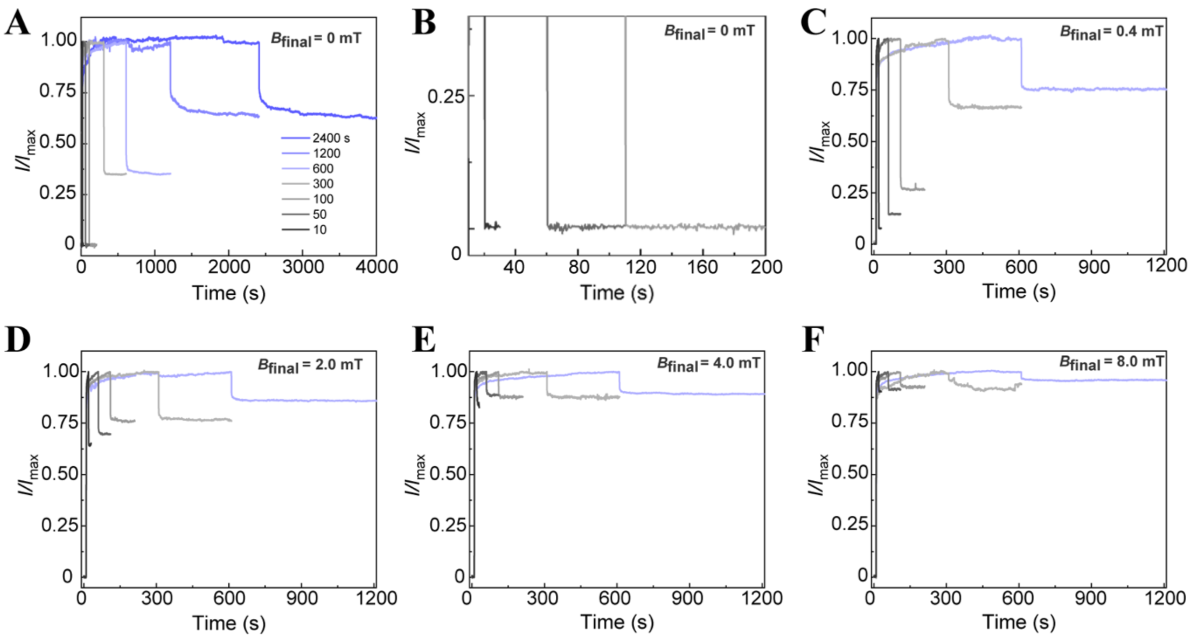

Fig. S18. The effect of exposure time at B_{set} = 20.0 mT on the residual normalized I, followed by reducing to various B_{final}. (**A**) B_{final} = 0.0 mT. (**B**) A higher-magnification view of fig. S18A in the range of 10-200 s. (**C**) B_{final} = 0.4 mT. (**D**) B_{final} = 2.0 mT. (**E**) B_{final} = 4.0 mT. (**F**) B_{final} = 8.0 mT. A summary is shown in fig. S19B. I_{max} is the current corresponding to the maximum available field of the electromagnet 40.2 mT.

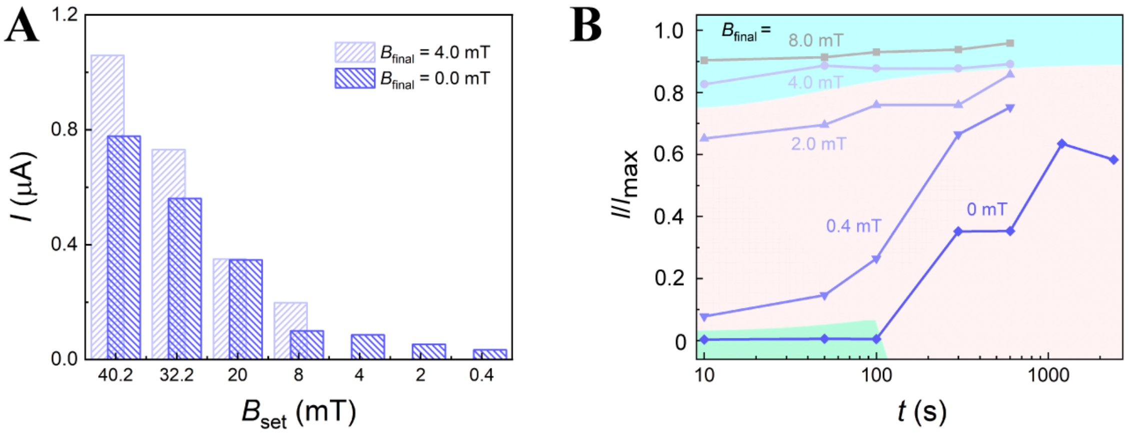

Fig. S19. Magnetic field and time dependent electric performances. (**A**) The residual current upon reducing the magnetic field from different B_{set} to B_{final} = 4.0 and 0 mT, respectively. (**B**) The effect of the exposure time at the initial field B_{set} = 20 mT on the residual current for different final fields B_{final}. Data are summarized from fig. S18. Note that 10 mg of the SFNCSs are sealed between two electrodes with a separation of 1.2 mm. I_{max} is the current corresponding to the maximum available field of the electromagnet 40.2 mT.

Dipole-dipole *vs.* gravitational interactions

By considering the particles as a spherical solid nickel with a density $\rho = 8.9$ g/cm^3 and combining the size $\bar{d}$ listed in table S2, the effective magnetization of each particle m_p can be estimated (see fig. S20, A and B) from the mass magnetization of the sample (M, fig. S7C) with Eq. 8:

$$m_p = M\frac{\pi\rho\bar{d}^3}{6\times10^{-3}}. \tag{9}$$

In the working range of the present electromagnet, i.e., 0 - 40.2 mT, the effective magnetic dipole-dipole interaction between two SFNCSs is in the order of 10^{-9} N (fig. S20, C and D). In comparison with gravity, the magnetic dipolar interaction between two particles is three orders of magnitude larger than the effect of gravity (fig. S20, E and F).

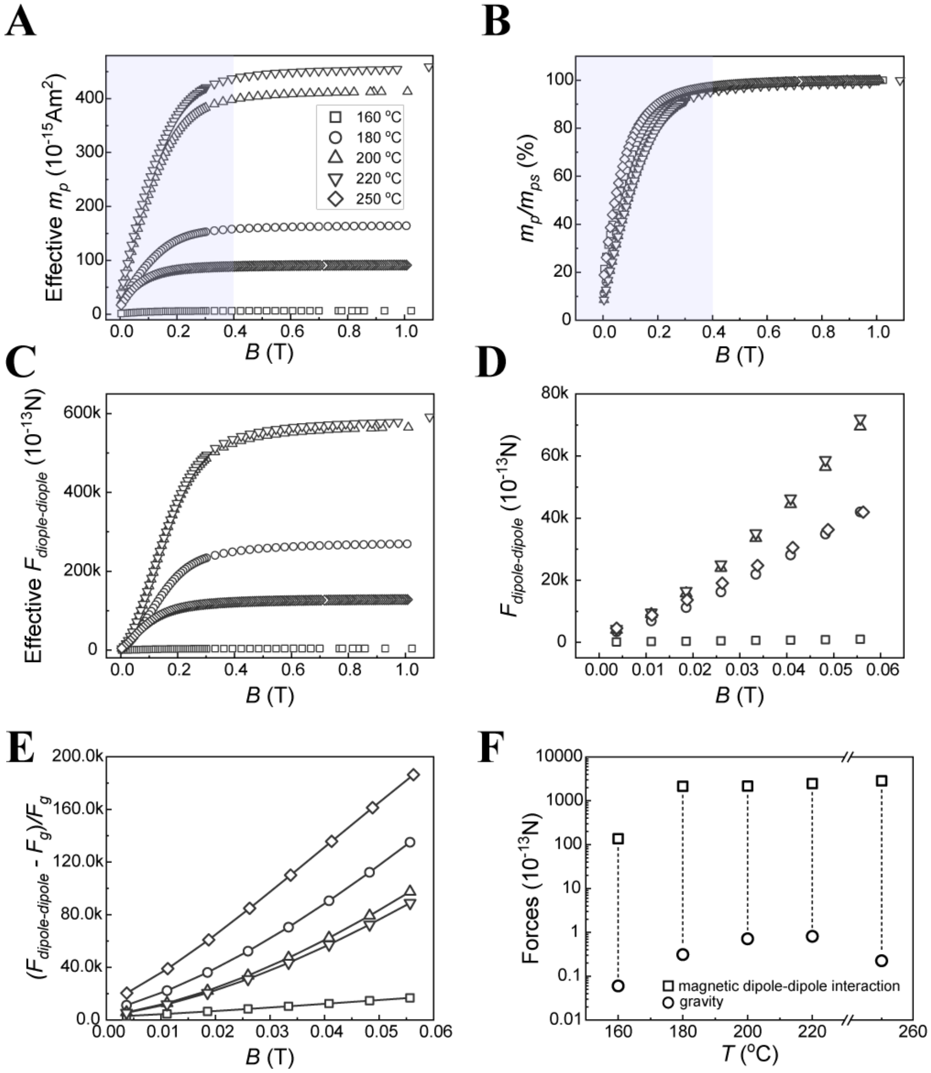

Fig. S20. The estimation of *B*-dependent magnetic dipolar interactions and gravitational forces. Magnetic remanent dipolar-dipolar interactions as a function of *B* and *T* in comparison with the gravitational force. The labels indicate the synthesis temperatures of SFNCSs.

Kinetic tuning of the *B*-driven bistable memory

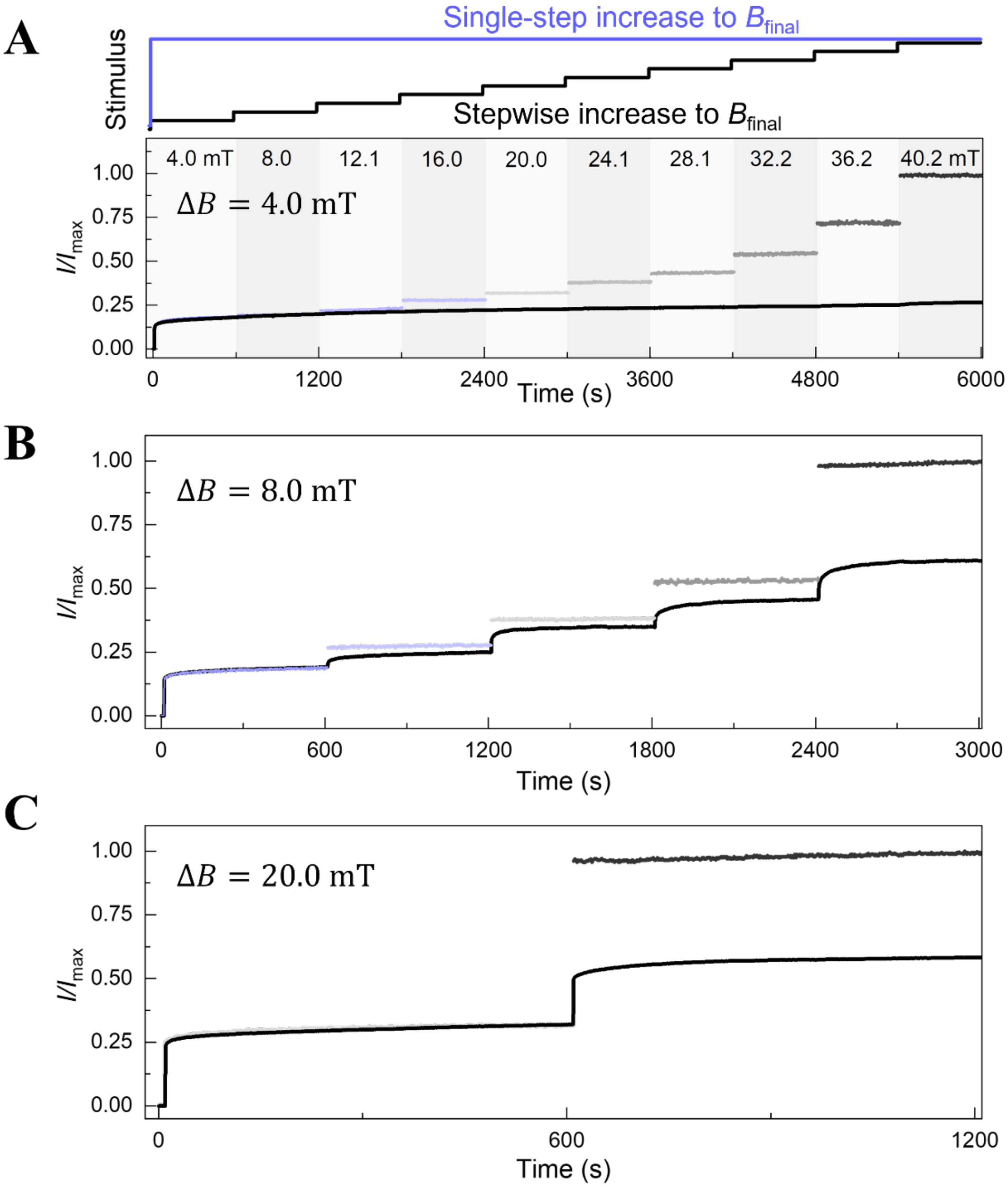

Fig. S21. Kinetic tuning of the *B*-driven bistable memory by using different steps to increase *B*. (**A** to **C**) Black line: The normalized *I* during the stepwise increase to B_{final} = 40.2 mT with a step-size ΔB of 4.0 mT in (A), 8.0 mT in (B) and 20.0 mT in (C), respectively. Colored line: The corresponding normalized *I* resulting from a using single-step increase to B_{final}. I_{max} is the current corresponding to the maximum available field of the electromagnet 40.2 mT.

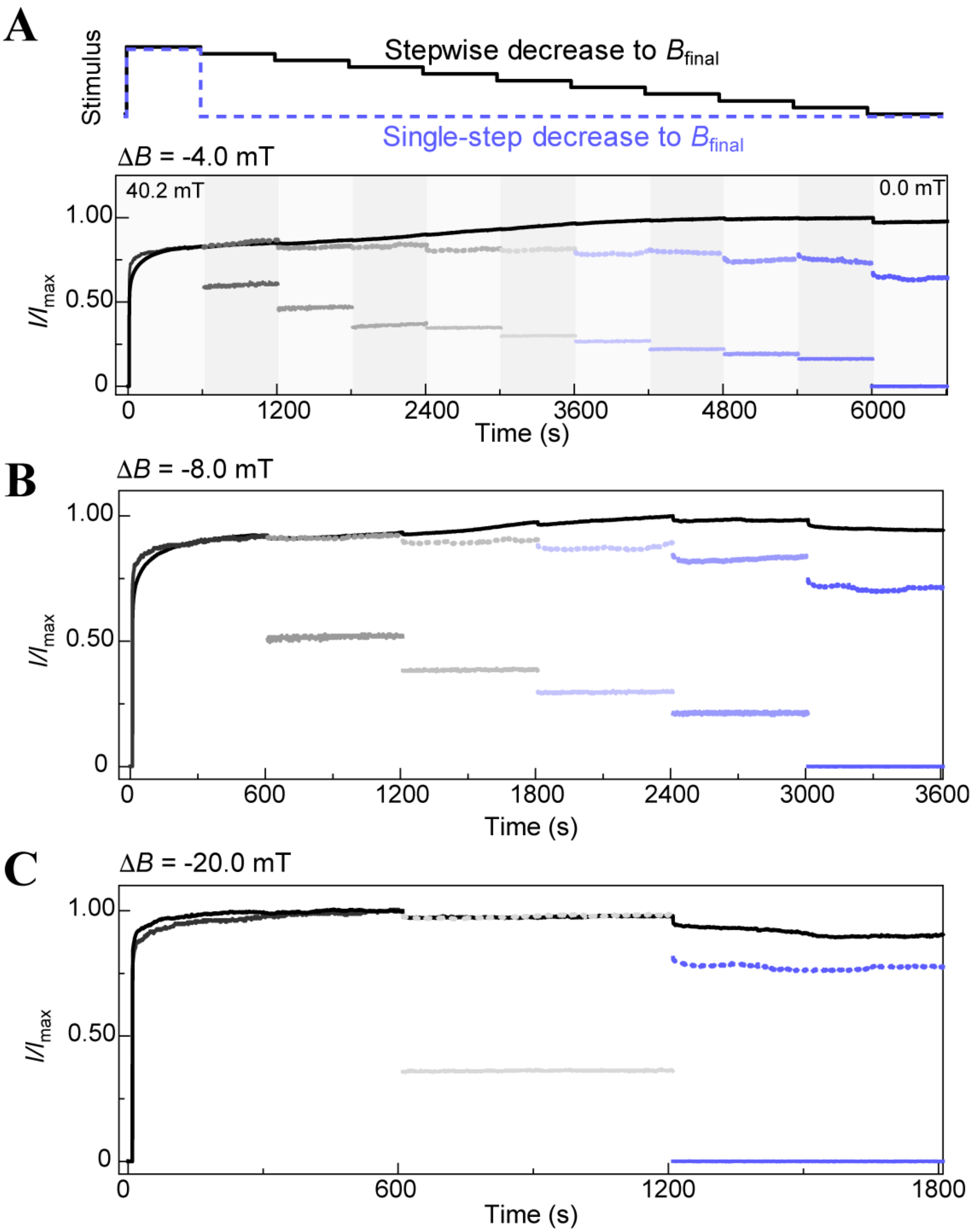

Fig. S22. Kinetic tuning of the *B*-driven bistable memory upon reducing the magnetic field from the initial value 40.2 mT at different steps. (**A** to **C**) Black line: The normalized *I* during stepwise decrease to B_{final} with a ΔB of -4.0 mT in (A), -8.0 mT in (B) and -20.0 mT in (C). Colored dash line: The normalized *I* that uses single-step decrease to B_{final}; Colored line: Normalized *I* based on single-step increase to B_{final}. I_{max} is the current corresponding to the maximum available field of the electromagnet 40.2 mT.

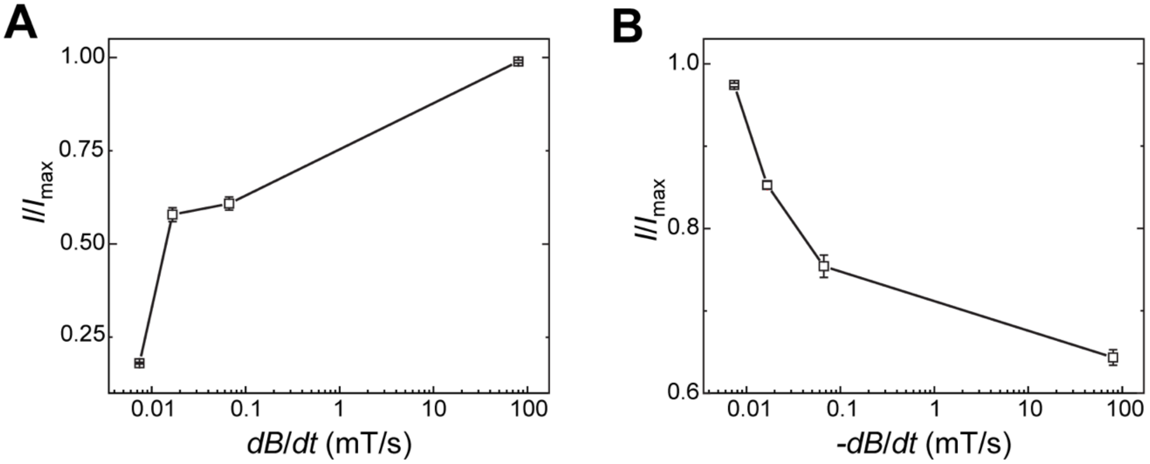

Fig. S23. The effect of average *B*-change rate *dB*/*dt* on I/I_{max}. The effect of *B*-change rate *dB*/*dt* on I/I_{max} in the processes of (A) increasing field from 0 to 40 mT and (B) decreasing field from 40 to 0 mT. I_{max} is the current corresponding to the maximum available field of the electromagnet 40.2 mT.

Magnetic-field pulsing-dependent plasticity

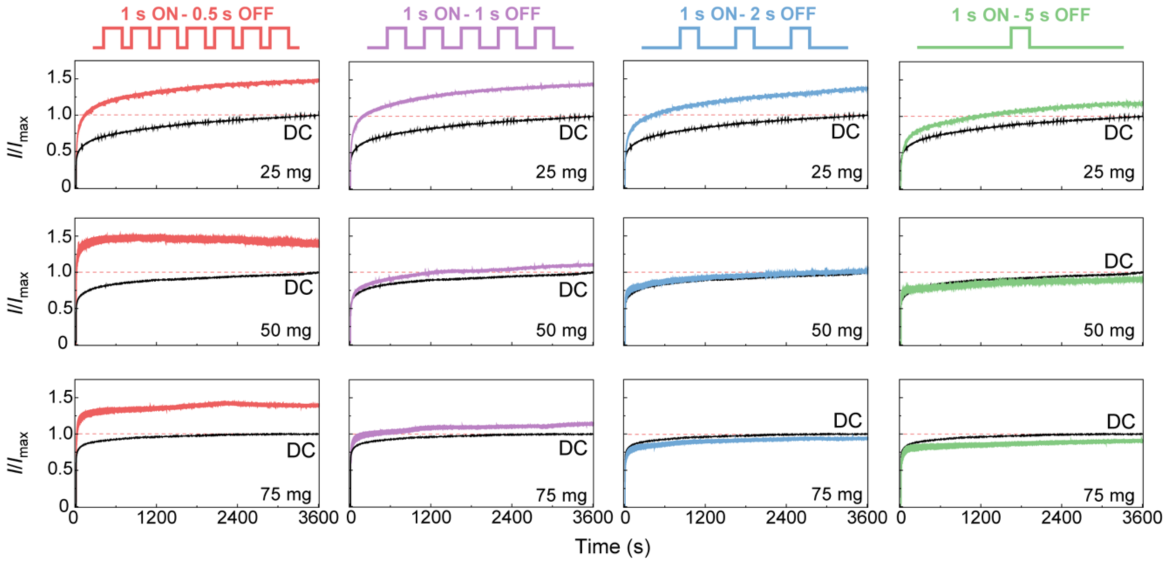

Fig. S24. Plasticity of electric current by repeating magnetic pulses. Colored lines: The normalized *I* upon applying periodic magnetic field pulses up to 32 mT using 25, 50, and 75 mg of SFNCSs using the electrode separation *d*=1.2 mm and electrode area 1.44 cm^2. The pulse ON-duration is fixed 1.0 s, whereas the OFF-durations are 0.5, 1.0, 2.0, and 5.0 s. Black lines: The corresponding normalized *I* using a DC magnetic field 32 mT. I_{max} is defined here as the current corresponding to 32 mT DC field. In some cases, the pulsed and DC cases overlap.

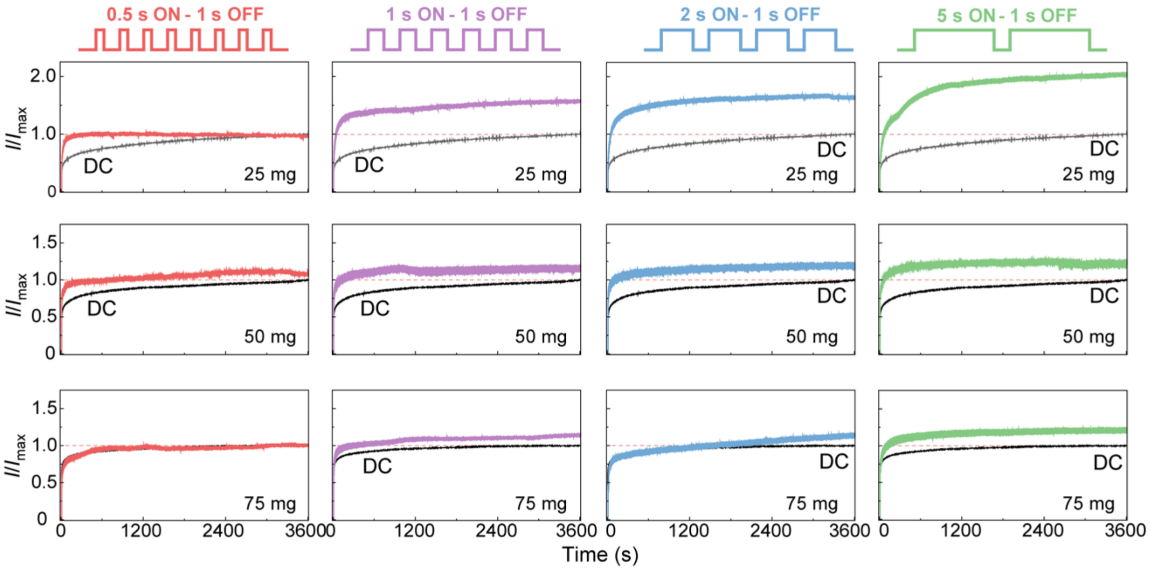

Fig. S25. Plasticity of electric current by repeating magnetic pulses. Colored lines: The normalized I upon applying periodic magnetic field pulses up to 32 mT using 25, 50, and 75 mg of SFNCSs using the electrode separation d=1.2 mm and electrode area 1.44 cm^2. The pulse ON-durations are 0.5, 1.0, 2.0, and 5.0 s, whereas the OFF-duration is fixed 1.0 s. Black lines: The corresponding normalized I using a DC magnetic field 32 mT. The I_{max} is defined here as the current corresponding to 32 mT DC field. In some cases, the pulsed and DC cases overlap.

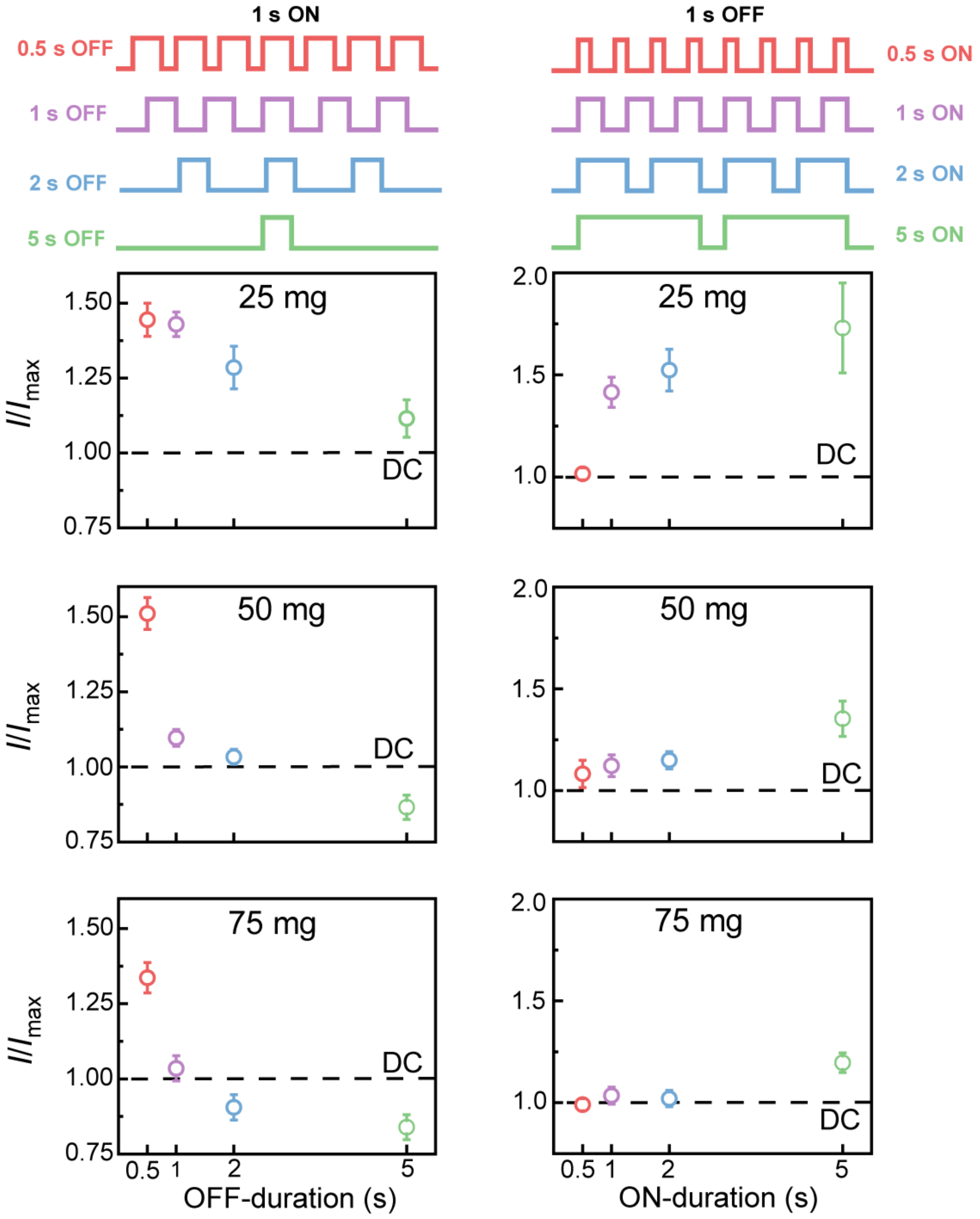

Fig. S26. Current plasticity upon magnetic field pulsing. Summarized results of figs. S24 and S25. The normalized I upon applying periodic magnetic field pulses up to 32 mT using 25, 50, and 75 mg of SFNCSs using the electrode separation d=1.2 mm and electrode area 1.44 cm^2. Dashed lines: The corresponding normalized I using a DC magnetic field 32 mT. The I_{max} is defined here as the current corresponding to 32 mT DC field.

Demonstration of switching plasticity using permanent magnets

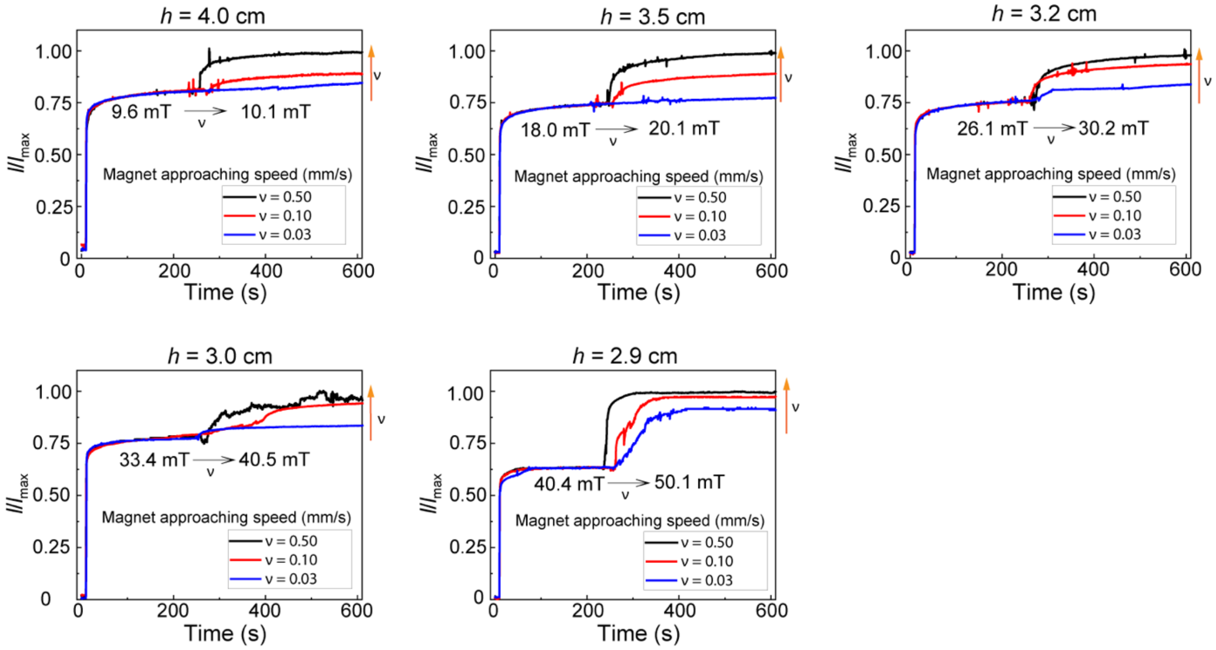

Fig. S27. Adaptive current plasticity by increasing the magnetic field when a permanent magnet approaches the electrodes slowly or rapidly using different velocities *v* at different heights *h* from the electrodes. Rapid approach leads to higher available current. The currents have been scaled in this case by the highest current observed in this experimentation, i.e., the most rapid approach speed. The arrows in the right highlight the effect of increasing approach speed.

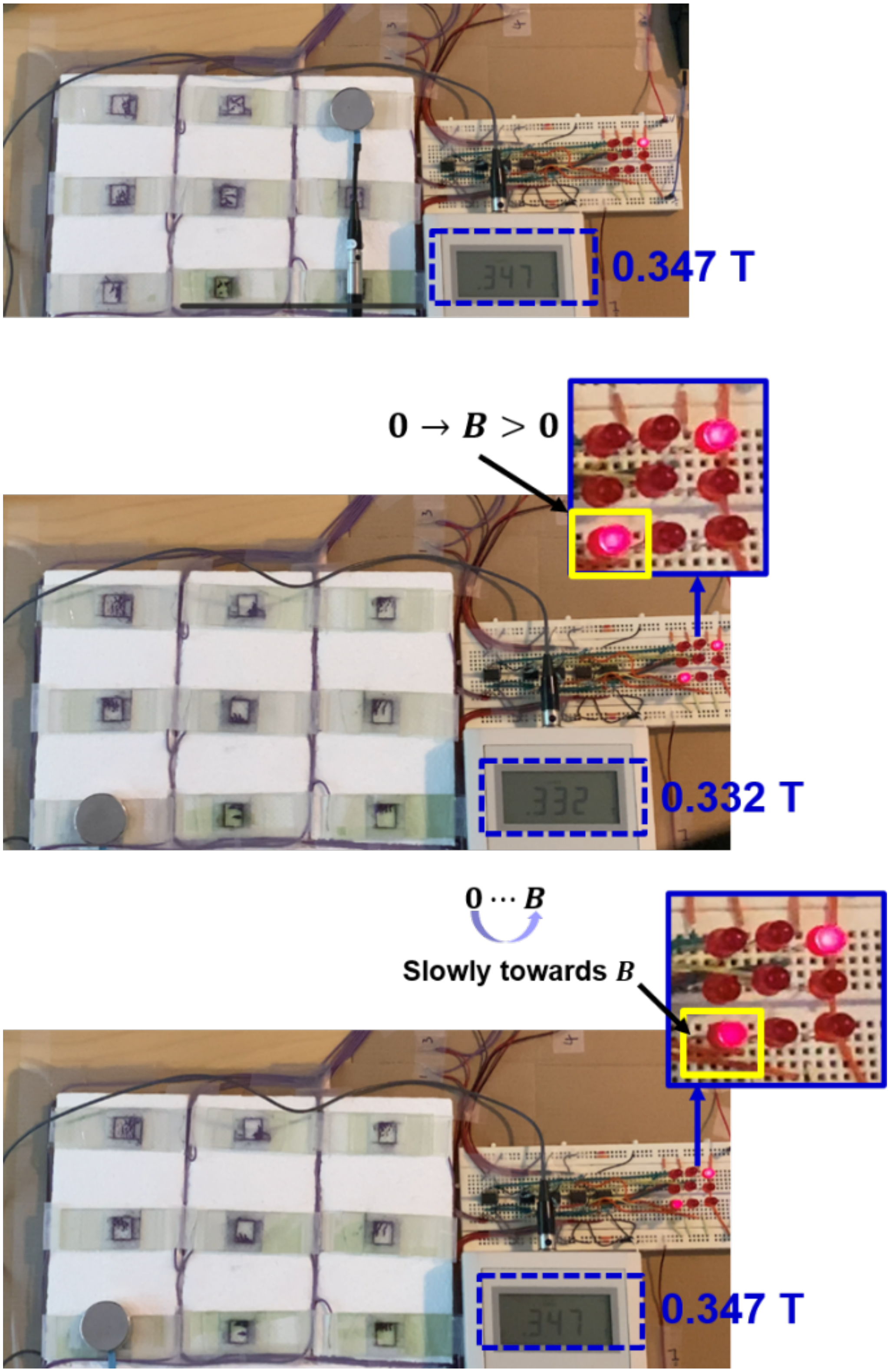

Fig. S28. Demonstration of the plasticity of LED brightness. Screenshots of movies S6 to S8, demonstrating the plasticity of LED brightness.

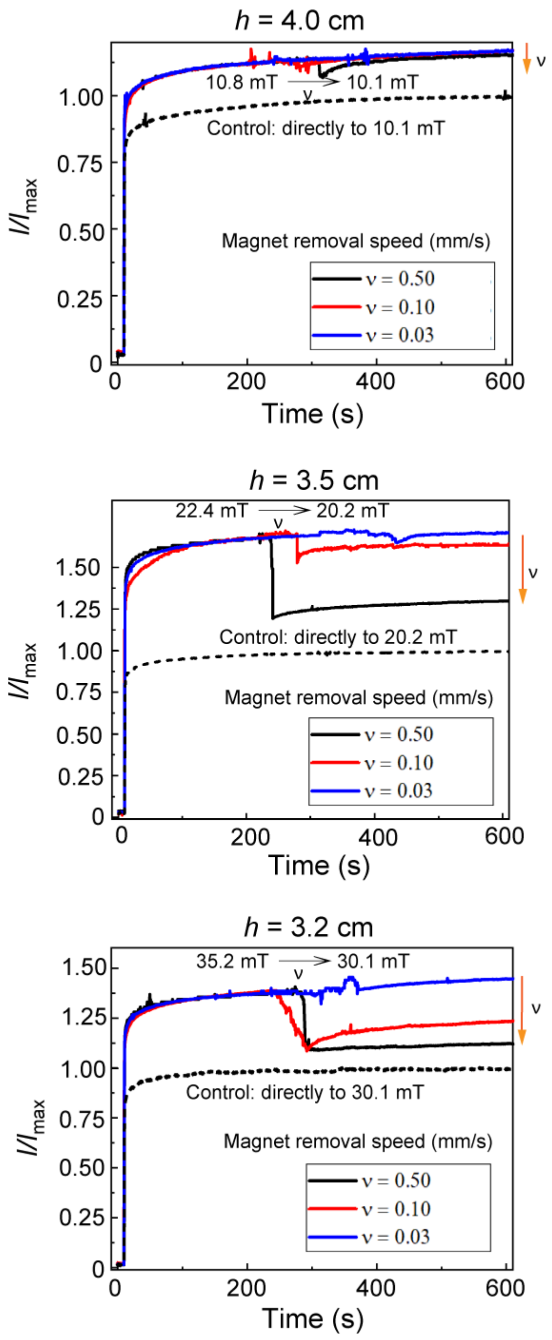

Fig. S29. Adaptive current plasticity by reducing the magnetic field when a permanent magnet is removed from the electrodes slowly or rapidly using different velocities *v* at different heights *h*. The currents have been scaled in this case by the current measured by directly applying the final field. Quick magnetic field removal leads to higher responses, i.e., lower currents, as highlighted by the arrows in the right.

Other Supplementary Materials include the following:

Movie S1. The top view of micropillar assembly at 40.2 mT and partial disassembly after this field is turned off. The particles are placed in a transparent chamber, facilitating the top view observation. Before the start of the movie S1, the field has been switched ON to 40.2 mT, whereafter the field was switched OFF as an initial preparation of the sample. Therefore, the present starting view shows this zero-field case, where the particles are distributed over the substrate with branched structures caused by the magnetic remanence of the particles. Then, the field is increased to 40.2 mT in a fraction of a second and the micropillars form. In the end, the field is removed and the pillars partially collapse and partially retain packing by colloidal jamming.

Movie S2. The side view of micropillar assembly at 40.2 mT and partial disassembly after this fields is turned off. Similar as movie S1 but showing the side view.

Movie S3. The top view of micropillar assembly and disassembly by applying pulsing B. The movie shows the top view of pillar formation and collapse with pulsing B in a transparent chamber. At the time 10 s of the movie, the pulsing magnetic field at 32 mT with $\Delta t_{\mathrm{ON}} = \Delta t_{\mathrm{OFF}} = 1.0$ s is applied. The retention of the pillared structures becomes pronounced upon continued application of B cycles.

Movie S4. The side view of micropillar assembly and disassembly by applying pulsing B. Similar as movie S3 but showing the side view.

Movie S5. Demonstration of using a permanent magnet for a memory. A permanent magnet rapidly approaches a sensor element allowing a bright LED ON. The subsequent removal of the magnet leaves the LED still ON due to the structural memory caused by colloidal jamming in micropillars. This also serves as a control to the demonstrations shown in movies S6 and S7.

Movie S6. Demonstration of using a permanent magnet to rapidly approach towards a sensor element. Immediately after movie S5, a magnet rapidly approaches towards another sensor element, leading to the LED ON with a same brightness as shown in movie S5.

Movie S7. Demonstration of using a permanent magnet to slowly approach towards a sensor element. Immediately after movie S5, a magnet slowly approaches towards another sensor element, leading to a dim LED ON. This low brightness of the LED is in contrast to that in movie S6.

Movie S8. Memory plasticity - Quickly remove the magnet. After rapidly approaching towards the sensor element and inducing a bright LED ON, a quick removal of the magnet decreases the brightness of the LED.

Movie S9. Memory plasticity - Slowly remove the magnet. After rapidly approaching towards the sensor element and inducing a bright LED ON, the subsequent slow removal of the magnet leads to a slight decrease of the brightness. In combination with movie S8, the memory plasticity of the LED brightness has been demonstrated by tuning the application manner of the magnet.

Movie S10. Demonstration of remote magnetic writing using LED arrays. Based on the structural memory as demonstrated in movie S5, remote writing a letter of ‘L’ on a 3 × 3 LED matrix is achieved with a magnet as a pen.

REFERENCES AND NOTES

1. P. Schattling, F. D. Jochum, P. Theato, Multi-stimuli responsive polymers–the all-in-one talents. *Polym. Chem.* **5**, 25–36 (2014).

2. M. W. Urban, *Stimuli-Responsive Materials: From Molecules to Nature Mimicking Materials Design* (Royal Society of Chemistry, 2016).

3. K. M. Herbert, S. Schrettl, S. J. Rowan, C. Weder, 50th anniversary perspective: Solid-state multistimuli, multiresponsive polymeric materials. *Macromolecules* **50**, 8845–8870 (2017).

4. X. Zhang, L. Chen, K. H. Lim, S. Gonuguntla, K. W. Lim, D. Pranantyo, W. P. Yong, W. J. T. Yam, Z. Low, W. J. Teo, H. P. Nien, Q. W. Loh, S. Soh, The pathway to intelligence: Using stimuli-responsive materials as building blocks for constructing smart and functional systems. *Adv. Mater.* **31**, 1804540 (2019).

5. Y. Xia, Y. He, F. Zhang, Y. Liu, J. Leng, A review of shape memory polymers and composites: Mechanisms, materials, and applications. *Adv. Mater.* **33**, 2000713 (2021).

6. C. Ma, S. Wu, Q. Ze, X. Kuang, R. Zhang, H. J. Qi, R. Zhao, Magnetic multimaterial printing for multimodal shape transformation with tunable properties and shiftable mechanical behaviors. *ACS Appl. Mater. Interfaces* **13**, 12639–12648 (2021).

7. F. Long, Y. Cheng, Y. Ren, J. Wang, Z. Li, A. Sun, G. Xu, Latest advances in development of smart phase change material for soft actuators. *Adv. Eng. Mater.* **24**, 2100863 (2021).

8. A. Lendlein, O. E. C. Gould, Reprogrammable recovery and actuation behaviour of shape-memory polymers. *Nat. Rev. Mater.* **4**, 116–133 (2019).

9. P. Ball, Coming alive, *Nat. Mater.* **20**, 285 (2021).

10. C. Kaspar, B. J. Ravoo, W. G. van der Wiel, S. V. Wegner, W. H. P. Pernice, The rise of intelligent matter. *Nature* **594**, 345–355 (2021).

11. A. Walther, Viewpoint: From responsive to adaptive and interactive materials and materials systems: A roadmap. *Adv. Mater.* **32**, 1905111 (2020).

12. H. Zhang, H. Zeng, A. Priimagi, O. Ikkala, Viewpoint: Pavlovian materials—Functional biomimetics inspired by classical conditioning. *Adv. Mater.* **32**, 1906619 (2020).

13. M. M. Lerch, A. Grinthal, J. Aizenberg, Viewpoint: Homeostasis as inspiration—Toward interactive materials. *Adv. Mater.* **32**, 1905554 (2020).

14. A. Halperin, M. Kröger, F. M. Winnik, Poly (N-isopropylacrylamide) phase diagrams: Fifty years of research. *Angew. Chem. Int. Ed.* **54**, 15342–15367 (2015).

15. W. Barthlott, M. Mail, B. Bhushan, K. Koch, Plant surfaces: Structures and functions for biomimetic innovations. *Nano-Micro. Lett.* **9**, 23 (2017).

16. B. Bhushan, Biomimetics: Lessons from nature - an overview. *Philos. Trans. R. Soc. A Math. Phys. Eng. Sci.* **367**, 1445–1486 (2009).

17. K. Autumn, Y. A. Liang, S. T. Hsieh, W. Zesch, W. P. Chan, T. W. Kenny, R. Fearing, R. J. Full, Adhesive force of a single gecko foot-hair. *Nature* **405**, 681–685 (2000).

18. B. Button, L. H. Cai, C. Ehre, M. Kesimer, D. B. Hill, J. K. Sheehan, R. C. Boucher, M. Rubinstein, A periciliary brush promotes the lung health by separating the mucus layer from airway epithelia. *Science* **337**, 937–941 (2012).

19. M. Seale, C. Cummins, I. M. Viola, E. Mastropaolo, N. Nakayama, Design principles of hair-like structures as biological machines. *J. R. Soc. Interface* **15**, 20180206 (2018).

20. D. R. McPherson, Sensory hair cells: An introduction to structure and physiology. *Integr. Comp. Biol.* **58**, 282–300 (2018).

21. I. Burgert, P. Fratzl, Actuation systems in plants as prototypes for bioinspired devices. *Philos. Trans. R. Soc. A Math. Phys. Eng. Sci.* **367**, 1541–1557 (2009).

22. J. J. Malicki, C. A. Johnson, The cilium: Cellular antenna and central processing unit. *Trends Cell Biol.* **27**, 126–140 (2017).

23. W. Gilpin, M. S. Bull, M. Prakash, The multiscale physics of cilia and flagella. *Nat. Rev. Phys.* **2**, 74–88 (2020).

24. A. Sidorenko, T. Krupenkin, A. Taylor, P. Fratzl, J. Aizenberg, Reversible switching of hydrogel-actuated nanostructures into complex micropatterns. *Science* **315**, 487–490 (2007).

25. B. A. Evans, A. R. Shields, R. L. Carroll, S. Washburn, M. R. Falvo, R. Superfine, Magnetically actuated nanorod arrays as biomimetic cilia. *Nano Lett.* **7**, 1428–1434 (2007).

26. A. del Campo, E. Arzt, Fabrication approaches for generating complex micro- and nanopatterns on polymeric surfaces. *Chem. Rev.* **108**, 911–945 (2008).

27. L. Qu, L. Dai, M. Stone, Z. Xia, Z. L. Wang, Carbon nanotube arrays with strong shear binding-on and easy normal lifting-off. *Science* **322**, 238–242 (2008).

28. B. Pokroy, S. H. Kang, L. Mahadevan, J. Aizenberg, Self-organization of a mesoscale bristle into ordered, hierarchical helical assemblies. *Science* **323**, 237–240 (2009).

29. J. V. I. Timonen, C. Johans, K. Kontturi, A. Walther, O. Ikkala, R. H. A. Ras, A facile template-free approach to magnetodriven, multifunctional artificial cilia. *ACS Appl. Mater. Interfaces* **2**, 2226–2230 (2010).

30. S. N. Khaderi, C. B. Craus, J. Hussong, N. Schorr, J. Belardi, J. Westerweel, O. Prucker, J. Rühe, J. M. J. den Toonder, P. R. Onck, Magnetically-actuated artificial cilia for microfluidic propulsion. *Lab Chip* **11**, 2002–2010 (2011).

31. X. He, M. Aizenberg, O. Kuksenok, L. D. Zarzar, A. Shastri, A. C. Balazs, J. Aizenberg, Synthetic homeostatic materials with chemo-mechano-chemical self-regulation. *Nature* **487**, 214–218 (2012).

32. D. Wang, Q. Sun, M. J. Hokkanen, C. Zhang, F. Y. Lin, Q. Liu, S. P. Zhu, T. Zhou, Q. Chang, B. He, Q. Zhou, L. Chen, Z. Wang, R. H. Ras, X. Deng, Design of robust superhydrophobic surfaces. *Nature* **582**, 55–59 (2020).

33. M. Sitti, D. T. Wiersma, Pros and cons: Magnetic versus optical microrobots. *Adv. Mater.* **32**, 1906766 (2020).

34. Y. Kim, X. Zhao, Magnetic soft materials and robots. *Chem. Rev.* **122**, 5317–5364 (2022).

35. M. Cui, T. Emrick, T. Russell, Stabilizing liquid drops in nonequilibrium shapes by the interfacial jamming of nanoparticles. *Science* **342**, 460–463 (2013).

36. Y. Wang, L. Li, D. Hofmann, J. E. Andrade, C. Daraio, Structured fabrics with tunable mechanical properties. *Nature* **596**, 238–243 (2021).

37. H. Bense, M. van Hecke, Complex pathways and memory in compressed corrugated sheets. *Proc. Natl. Acad. Sci. U.S.A.* **118**, e2111436118 (2021).

38. T. Chen, M. Pauly, P. M. Reis, A reprogrammable mechanical metamaterial with stable memory. *Nature* **589**, 386–390 (2021).

39. J. Frenkel, J. Doefman, Spontaneous and induced magnetisation in ferromagnetic bodies. *Nature* **126**, 274–275 (1930).

40. B. Peng, X. Zhang, D. G. A. L. Aarts, R. P. A. Dullens, Superparamagnetic nickel colloidal nanocrystal clusters with antibacterial activity and bacteria binding ability. *Nat. Nanotechnol.* **13**, 478–482 (2018).

41. Y. Chai, A. Lukito, Y. Jiang, P. D. Ashby, T. P. Russell, Fine-tuning nanoparticle packing at water-oil interfaces using ionic strength. *Nano Lett.* **17**, 6453–6457 (2017).

42. P. Y. Kim, Y. Gao, Z. Fink, A. E. Ribbe, D. A. Hoagland, T. P. Russell, Dynamic reconfiguration of compressed 2D nanoparticle monolayers. *ACS Nano* **16**, 5496–5506 (2022).

43. H. Ikeda, C. Brito, M. Wyart, F. Zamponi, Jamming with tunable roughness. *Phys. Rev. Lett.* **124**, 208001 (2020).

44. F. Xiong, P. Wang, A. H. Clark, T. Bertrand, N. T. Ouellette, M. D. Shattuck, C. S. O'Hern, Comparison of shear and compression jammed packings of frictional disks. *Granul. Matter* **21**, 1–14 (2019).

45. H. Zhang, M. Widom, Field-induced forces in colloidal particle chains. *Phys. Rev. E* **51**, 2099–2103 (1995).

46. T. C. Halsey, A. J. Levine, How sandcastles fall. *Phys. Rev. Lett.* **80**, 3141–3144 (1998).

47. E. R. Kandel, *In Search of Memory: The Emergence of a New Science of Mind* (W. W. Norton & Co., 2006).

48. J. Yu, B. Wang, X. Du, Q. Wang, L. Zhang, Ultra-extensible ribbon-like magnetic microswarm. *Nat. Commun.* **9**, 3260 (2018).

49. W. Hu, G. Z. Lum, M. Mastrangeli, M. Sitti, Small-scale soft-bodied robot with multimodal locomotion. *Nature* **554**, 81–85 (2018).

50. R. E. Rosensweig, *Ferrohydrodynamics* (Cambridge Univ. Press, 1985).
